# Direct synthesis of p-type PbS quantum dot inks toward wafer-scale SWIR imaging

**DOI:** 10.1093/nsr/nwag421

**Published:** 2026-07-11

**Authors:** Yang Liu, Shengyu Ma, Chengjie Deng, Chunmeng Liu, Yu Yin, Kunyuan Lu, Guozheng Shi, Lin Yuan, Yao Yin, Juncheng Zhu, Xinyang Huang, Lizhen Huang, Jianlong Xu, Liang Gao, Zeke Liu, Wanli Ma

**Affiliations:** State Key Laboratory of Bioinspired Interfacial Materials Science, Institute of Functional Nano & Soft Materials (FUNSOM), Soochow University, Suzhou 215123, China; State Key Laboratory of Bioinspired Interfacial Materials Science, Institute of Functional Nano & Soft Materials (FUNSOM), Soochow University, Suzhou 215123, China; Wuhan National Laboratory for Optoelectronics (WNLO) and School of Optical and Electronic Information, Huazhong University of Science and Technology, Wuhan 430070, China; Division of Physical Science and Engineering, Center for Renewable Energy and Storage Technologies (CREST), King Abdullah University of Science and Technology, Thuwal 23955-6900, Saudi Arabia; State Key Laboratory of Bioinspired Interfacial Materials Science, Institute of Functional Nano & Soft Materials (FUNSOM), Soochow University, Suzhou 215123, China; State Key Laboratory of Bioinspired Interfacial Materials Science, Institute of Functional Nano & Soft Materials (FUNSOM), Soochow University, Suzhou 215123, China; State Key Laboratory of Bioinspired Interfacial Materials Science, Institute of Functional Nano & Soft Materials (FUNSOM), Soochow University, Suzhou 215123, China; State Key Laboratory of Bioinspired Interfacial Materials Science, Institute of Functional Nano & Soft Materials (FUNSOM), Soochow University, Suzhou 215123, China; State Key Laboratory of Bioinspired Interfacial Materials Science, Institute of Functional Nano & Soft Materials (FUNSOM), Soochow University, Suzhou 215123, China; State Key Laboratory of Bioinspired Interfacial Materials Science, Institute of Functional Nano & Soft Materials (FUNSOM), Soochow University, Suzhou 215123, China; State Key Laboratory of Bioinspired Interfacial Materials Science, Institute of Functional Nano & Soft Materials (FUNSOM), Soochow University, Suzhou 215123, China; State Key Laboratory of Bioinspired Interfacial Materials Science, Institute of Functional Nano & Soft Materials (FUNSOM), Soochow University, Suzhou 215123, China; State Key Laboratory of Bioinspired Interfacial Materials Science, Institute of Functional Nano & Soft Materials (FUNSOM), Soochow University, Suzhou 215123, China; Wuhan National Laboratory for Optoelectronics (WNLO) and School of Optical and Electronic Information, Huazhong University of Science and Technology, Wuhan 430070, China; State Key Laboratory of Bioinspired Interfacial Materials Science, Institute of Functional Nano & Soft Materials (FUNSOM), Soochow University, Suzhou 215123, China; State Key Laboratory of Bioinspired Interfacial Materials Science, Institute of Functional Nano & Soft Materials (FUNSOM), Soochow University, Suzhou 215123, China

**Keywords:** quantum dot, SWIR imaging, ink-processed devices, pixel-to-pixel uniformity

## Abstract

PbS quantum dots (QDs) are highly promising for scalable short-wave infrared (SWIR) optoelectronics due to their tunable bandgap and solution-processability. While efficient devices require well-defined p-n homojunctions, the fabrication of p-type QD layers remains a critical bottleneck compared with the well-established n-type QD inks. Current fabrication of p-type QD layers relies on solid-state ligand exchange (SSLE). However, SSLE suffers from incomplete ligand replacement and non-uniform oxidation, causing film inhomogeneity that prevents wafer-scale manufacturing. Here, we report a direct synthesis of p-type PbS QD inks with tunable QD size, optical absorption, and p-type electronic characteristics. The resulting inks yield conductive p-type films on 6-inch Si substrates with highly uniform thickness. Using this strategy, we realize SWIR photodetectors with fully ink-processed PbS QD p-n junctions, together with a prototype imaging module. When integrated with a recognition algorithm, the imager provides SWIR images that facilitate vehicle recognition under foggy conditions. This work establishes a scalable route toward future high-performance, wafer-level SWIR optoelectronic platforms.

## Teaser

Directly synthesized p-type quantum dot inks enable uniform, high-quality short-wave infrared imaging devices, offering a scalable path toward lower-cost infrared cameras for imaging and intelligent sensing.

## INTRODUCTION

Short-wave infrared (SWIR) technology possesses unique advantages in biomedical imaging, all-weather environmental monitoring, and chemical identification [1,2]. The rapid development in smartphones, biometric systems, and autonomous vehicles has driven demand for cost-effective SWIR materials [3], while conventional SWIR semiconductors (InGaAs, CdHgTe, etc.) are extremely expensive due to the complex fabrication and heterogeneous integration processes [4,5]. PbS quantum dots (QDs), as an emerging class of solution-processable semiconductor materials, have become promising candidates for the development of near-infrared and SWIR optoelectronic devices [6–12], due to their tunable optical absorption, high carrier mobility, and multiple exciton generation effects [13–15]. Furthermore, the solution processability is compatible with large-scale manufacturing techniques such as spin-coating and printing [16,17], and more importantly, enables monolithic integration with standard silicon-based readout circuits, thereby offering a practical and scalable technological pathway toward wafer-level, low-cost infrared imaging chips [18–21].

Large-area infrared photodetectors require spatially uniform electronic junctions or built-in fields for efficient carrier separation and consistent pixel response. In mature InGaAs/InP detectors, p-type regions are defined by established wafer-scale doping or diffusion processes. In contrast, in emerging solution-processed and low-dimensional semiconductors, such as colloidal QDs and two-dimensional materials, electronic polarity and junction formation are often governed by surface chemistry, interfaces, contact-induced doping, or post-treatment processes [14,22–24]. Therefore, the surface-governed electronic properties of PbS QDs become increasingly important when devices are scaled from small-area photodiodes to wafer-level imaging arrays.

The efficient PbS QD optoelectronic devices typically require the construction of p-n junctions [24–27]. At present, conductive n-type PbS QD films are comparatively well developed, with established methods such as solution-phase ligand exchange (SPLE) and the direct synthesis of conductive QD inks [26–34]. In contrast, the fabrication of conductive p-type QD films remains heavily dependent on solid-state ligand exchange (SSLE) using thiol-based ligands [24–29,35,36]. However, it is known that SSLE induces non-uniform ligand exchange and volumetric contraction, which causes film cracking [37,38]. Moreover, the oxidation process required to activate p-type doping further amplifies the intrinsic energetic inhomogeneity of QD solids. Incomplete ligand exchange, heterogeneous QD fusion, residual surface species, and non-uniform inter-dot coupling can lead to spatially uneven oxidation kinetics and trap formation, thereby broadening the local energetic landscape and aggravating electronic disorder across the film [26,39]. Consequently, these limitations severely compromise device reproducibility and render SSLE processes incompatible with scalable wafer-level manufacturing. A few studies have reported p-type PbS QD inks via SPLE; however, the process generally relies on complex multistep exchange procedures and has thus far been limited primarily to small-area device demonstrations [40–42]. Therefore, developing efficient and scalable strategies for directly synthesized p-type PbS QD inks is crucial for future wafer-scale device fabrication.

In this work, we employ Pb(SCN)_2_ as the lead precursor and introduce para-substituted thiol ligands to directly synthesize p-type PbS QDs with tunable p-type electronic characteristics. Through a seeded-growth strategy, the first-excitonic absorption peak of the QDs is extended from ~1100 nm to beyond 2000 nm. Leveraging *in-situ* thiolate passivation, the resulting QD inks exhibit uniform surface structure and electronic distribution, enabling the uniform deposition of homogeneous conductive p-type PbS QD films on 6-inch Si substrates without any ligand-exchange step. Consequently, fully ink-processed PbS QD p-n junction photodetectors and prototype imaging modules based on these inks enable stable and spatially uniform image acquisition. Furthermore, by integrating recognition algorithms, the system reliably performs vehicle detection under both sunny and foggy conditions. These results provide a foundation for scalable and intelligent SWIR imaging technologies based on uniform large-area QD films, toward wafer-level integration.

## RESULTS

### p-type QD inks

We previously reported the direct synthesis of n-type QD inks, where PbI_2_ and Diphenylthiourea (DPhTA) were dissolved in *N,N*-dimethylformamide (DMF), followed by the injection of butylamine (BA) to trigger the reaction [43]. For the synthesis of p-type QDs, the commonly used lead precursor PbI_2_ cannot be used since the introduction of halide ligands leads to n-type behavior [14,26]. We thus evaluated alternative lead salts, including Pb(SCN)_2_, PbCO_3_, PbSO_4_, and Pb(Ac)_2_. Among these, only Pb(SCN)_2_ exhibited good solubility in DMF (Fig. S1). We then substituted PbI_2_ with Pb(SCN)_2_ to conduct the synthesis of p-type QDs. However, when Pb(SCN)_2_ was used as the sole lead precursor, the obtained materials were unable to form a stable dispersion in DMF. The solution appeared gray, and the solid was easily precipitated by low-speed centrifugation without the use of an antisolvent (Fig. S2a and b). The X-ray diffraction (XRD) pattern (Fig. S3) confirms that the obtained solid corresponds to the cubic phase of PbS without any other phase. The transmission electron microscopy (TEM) image reveals poor size uniformity and severe aggregation, with large, irregular clusters distributed throughout the sample (Fig. S2c).

**Figure 1.**
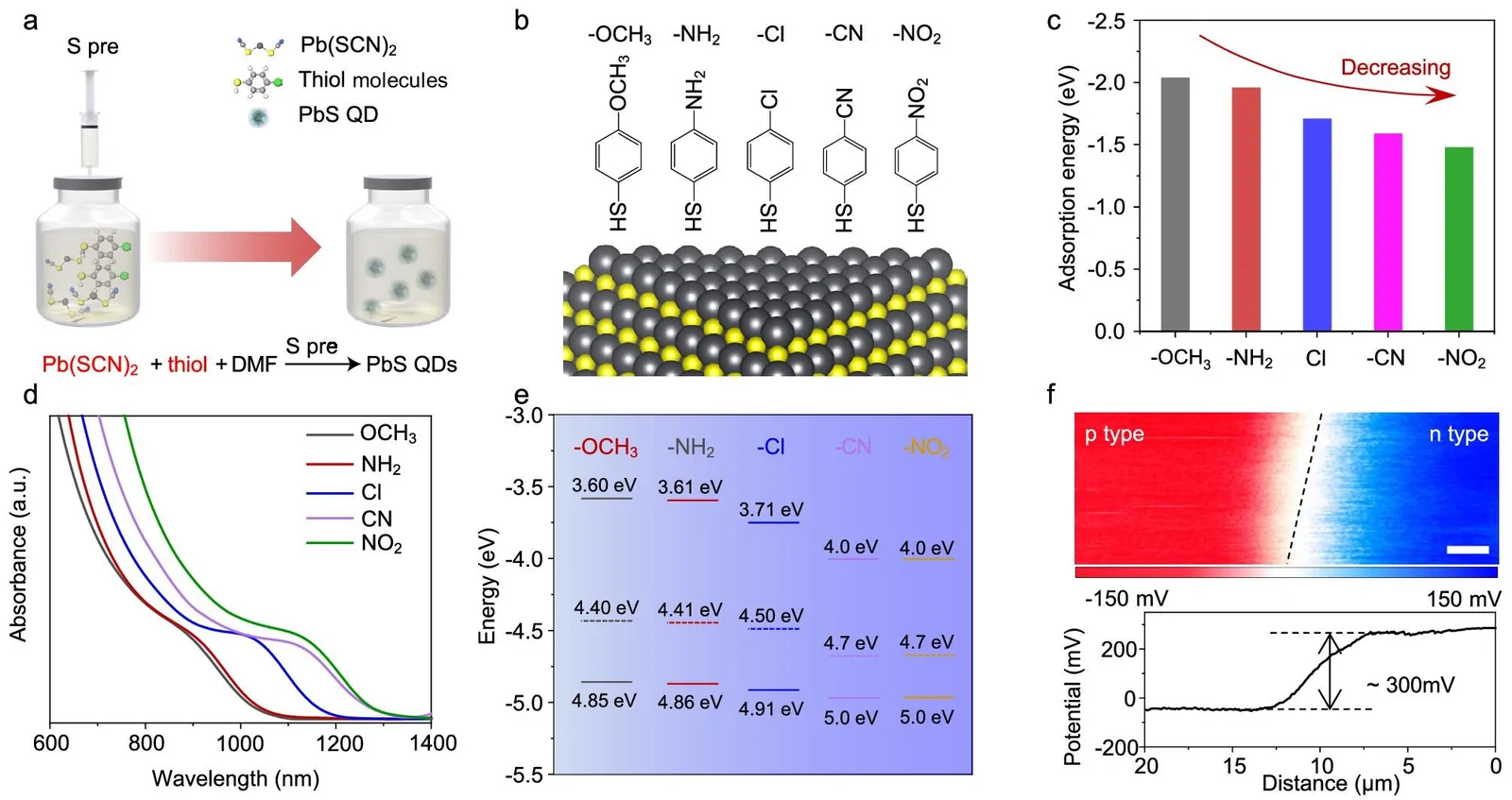
(a) Schematic of the synthesis of p-type PbS QDs. (b) Schematic structure of the thiol ligands. (c) Adsorption energy of molecules on the surface of PbS (111) plane. (d) Absorbance spectra of PbS QDs synthesized with different molecules. (e) Energy levels of PbS QDs with different thiol ligands. (f) The KPFM surface potential mapping (top, scale bar 2 μm), and the surface potential variations of the p-n homojunction QD film (bottom).

To address this issue, Pb(SCN)_2_ was dissolved in DMF as the Pb precursor, while thiol-containing small molecules were introduced to regulate the reaction kinetics and suppress QD aggregation. DPhTA dissolved in BA was subsequently injected as the S precursor, enabling the successful synthesis of well-dispersed p-type PbS QDs (Fig. 1a). Figure 1b shows the chemical structures of various functional small molecules attached to the (111) plane of the PbS QDs, including 4-methoxybenzenethiol (−OCH_3_), 4-aminobenzenethiol (−NH_2_), 4-chlorothiophenol (−Cl), 4-mercaptobenzonitrile (−CN), and 4-nitrothiophenol (−NO_2_). The electron-withdrawing capabilities of the para-substituents follow the order: −OCH_3_ < −NH_2_ < −Cl < −CN < −NO_2_. This trend is corroborated by the electrostatic potential mapping, which reveals a corresponding decrease in the negative charge of the thiol group as the electron-withdrawing strength of the substituents increases (Fig. S4). To elucidate the ligand–surface interactions, we calculated the adsorption energies of the small molecules on the PbS (111) surface. All the density functional theory calculations were performed with the DS-PAW package [44], using the Perdew-Burke-Ernzerhof functional within the generalized gradient approximation [45]. The calculated adsorption energies become less negative as the electron-withdrawing ability increases, indicating weakened ligand–surface binding (Fig. 1c and Fig. S5). Specifically, the −OCH_3_ group exhibits the strongest adsorption, while the −NO_2_ group shows the weakest adsorption. Ligand adsorption strength strongly influences QD growth during synthesis.

Absorbance and PL measurements reveal that both the absorbance and PL spectra of the QDs redshift as the electron-withdrawing capability of the substituents increases (Fig. 1d and Fig. S6). We hypothesize that this phenomenon may be attributed to the binding energy of thiol groups on the QD surface, which affects the diffusion and growth behavior of precursors toward the surface during QD formation. Specifically, stronger electron‑withdrawing groups weaken thiol binding to the QD surface, thereby facilitating precursor diffusion and promoting QD growth, ultimately increasing QD size [46]. The XRD data (Fig. S7) support this observation, demonstrating that the progressive reduction in FWHM (reflecting QD size growth) correlates with the substituents’ enhanced electron-withdrawing ability, consistent with the absorbance and PL trends. To further investigate the morphological and size variations of the QDs synthesized with different ligands, TEM characterization was conducted (Fig. S8). The results revealed that the PbS QDs exhibited good dispersion and size uniformity, with larger QDs observed for ligands with stronger electron-withdrawing groups.

To investigate the energy levels of the obtained QDs, ultraviolet photoelectron spectroscopy measurements were performed. The p-type PbS QDs were successfully synthesized using Pb(SCN)_2_ and small thiol ligands (Fig. 1e and Fig. S9), exhibiting a progressive decrease in the energy offset between the valence band maximum and the Fermi level, from 0.45 eV (−OCH_3_) to 0.45 eV (−NH_2_), 0.41 eV (−Cl), 0.30 eV (−CN), and 0.30 eV (−NO_2_). It is evident that the energy offset gradually decreases as the electron-withdrawing capability of the substituents increases. This phenomenon may be attributed to the effect of the para-substituents on the negative charge of the thiol group, which modifies the coupling interaction between the ligands and the QDs, thereby influencing the doping behavior of the QDs [14]. Surface potential differences between conductive n-type and p-type PbS QD films were also characterized using Kelvin probe force microscopy (KPFM). The contact potential measurements revealed an ~300 mV potential difference between the conductive n-type and p-type QD films, confirming the energy shift of the Fermi level between the two types of QDs within the films (Fig. 1f).

### Size-tunable QD inks

**Figure 2.**
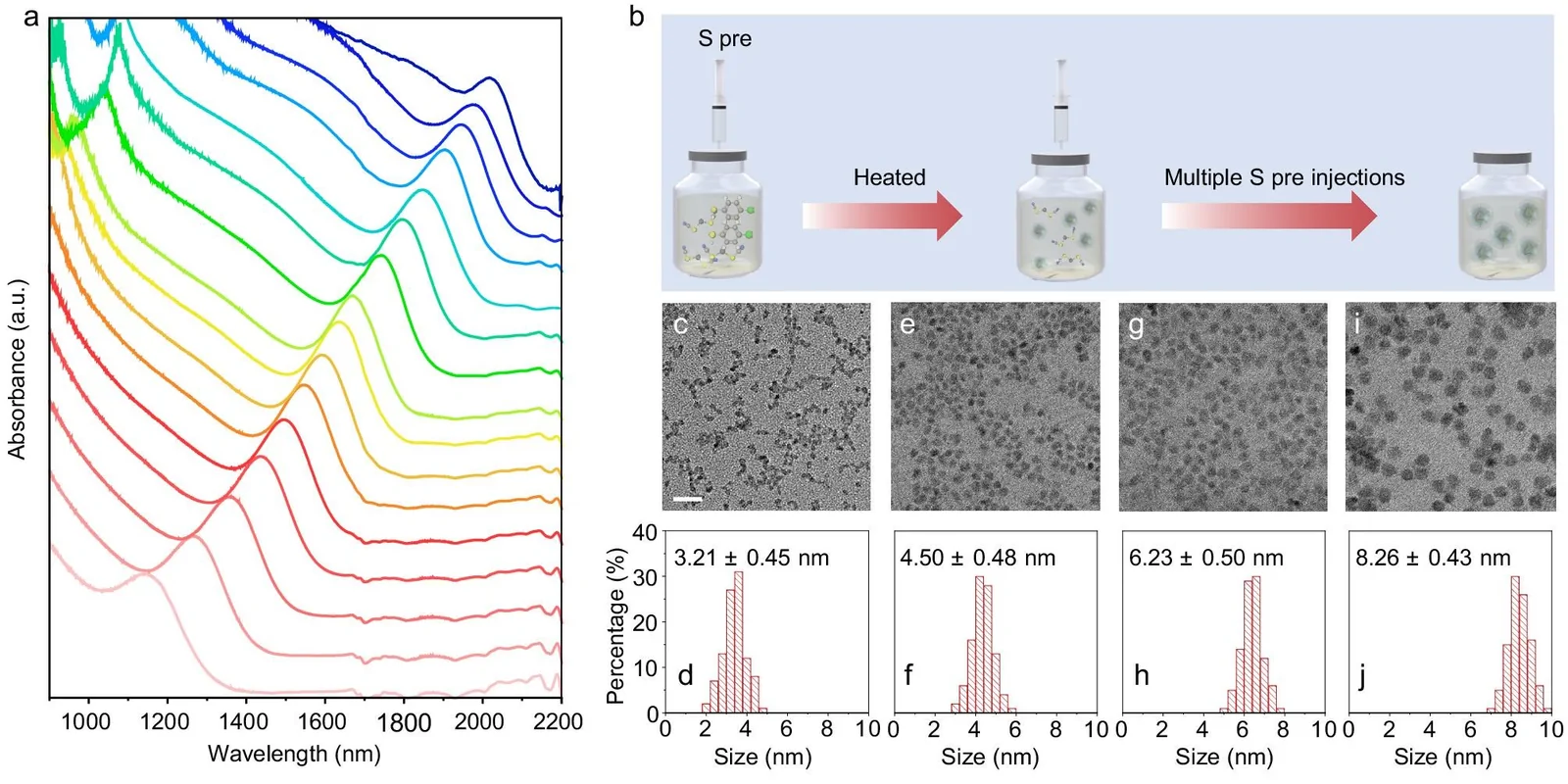
(a) Absorbance spectra of p-type PbS QDs with different first-excitonic absorption peaks. (b) Schematic illustration of the size-tunable synthesis of PbS QDs. (c, e, g, i) TEM images of p-type QDs with first-excitonic absorption peaks at 1100, 1410, 1800, and 2020 nm, respectively. (d, f, h, j) The corresponding size distribution histograms. Scale bar: 20 nm.

During the synthesis of p-type PbS QD inks, the QDs maintained good uniformity and were effectively stabilized by the small thiol ligands. To obtain the size-tunable PbS QD inks, we first synthesized PbS seeds, followed by a gradual injection of sulfur precursors to obtain larger PbS QDs (Fig. 2b). Initially, Pb(SCN)_2_ and 4-Mercaptobenzonitrile were dissolved in DMF, followed by the injection of sulfur precursors into the solution to form the PbS seeds. In the second step, the sulfur precursor was gradually injected into the seeds, with careful control of the precursor amount at each injection step to promote the growth of PbS QDs while preventing nucleation (Fig. S10). We then successfully synthesized PbS QD inks with first-excitonic absorption peaks beyond 2000 nm (Fig. 2a). TEM images (Fig. 2c–j) confirmed the controllable and tunable average QD sizes ranging from ~3.2 nm to above 8.2 nm, with regular spherical shapes. XRD measurements of the different-sized PbS QDs confirmed that the QD crystal structure is consistent with the cubic phase (Fig. S11). Furthermore, we investigated the relationship between the concentration of the sulfur precursor during the subsequent injection and the QD size (Figs S12 and S13). As the sulfur precursor concentration increases during multiple injections, the QDs become smaller and less uniform, with TEM images revealing the emergence of smaller QDs under high sulfur concentrations (Fig. S14), consistent with secondary nucleation driven by elevated monomer supersaturation that suppresses growth on pre-existing QD surfaces [47,48].

### SWIR photodetector and imaging

**Figure 3.**
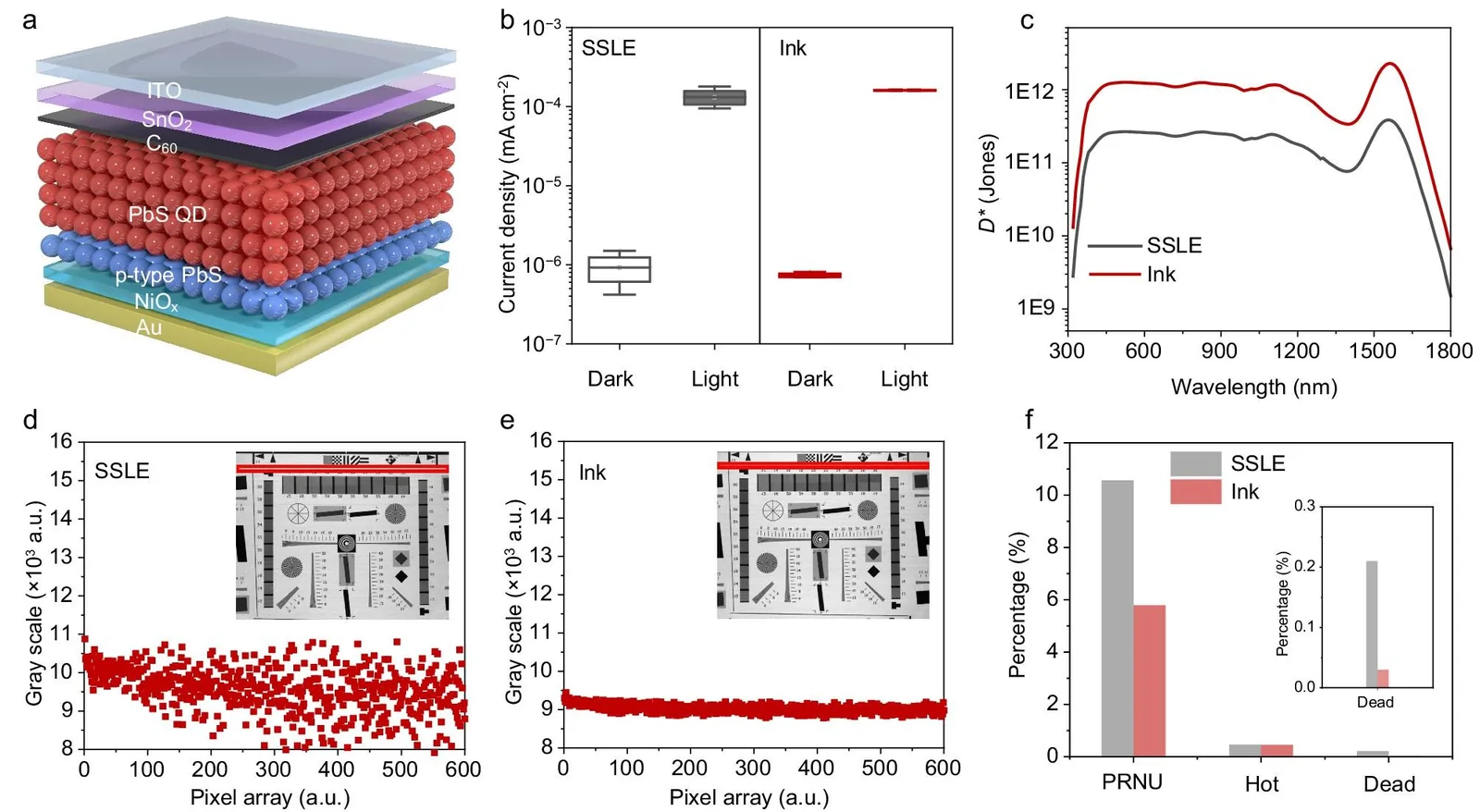
(a) Device architecture of the PbS QD photodiode. (b, c) Statistical comparison of the dark and light current density (b), and specific detectivity (c) of SSLE- and ink-based devices. (d, e) Pixel-wise grayscale values extracted from the ISO 12233 test chart, SSLE- (d) and ink-based imagers (e). Inset: the ISO 12233 test chart captured by the QD imager to measure the spatial frequency response. (f) Comparison of photoresponse non-uniformity (PRNU), hot pixels, and dead pixels between SSLE- and ink-based imagers.

Having established the controlled synthesis and uniformity of the p-type PbS QD inks, we next investigated their performance as the hole transport layer (HTL) in SWIR photodetectors. In state-of-the-art PbS QD photodiodes, the HTL is typically fabricated via 1,2-ethanedithiol (EDT)-based SSLE. Photodiodes with a device architecture of Au/NiO_*x*_/p-type PbS QDs/n-type PbS QDs/C_60_/SnO_2_/ITO were fabricated (Fig. 3a). The p-type PbS ink used for device fabrication was synthesized using −CN as the ligand. The ink-based devices exhibit key performance metrics comparable to those of the SSLE-based devices, demonstrating that the ink-based approach can serve as an effective alternative to conventional SSLE-based HTLs (Fig. 3b and c). In addition, device-to-device reproducibility statistics for independently fabricated devices (Fig. 3b and Fig. S15) further support the high reproducibility of the device fabrication process. To comprehensively evaluate device performance, external quantum efficiency, responsivity, noise current spectra, response speed and linear dynamic range were measured (Figs S16–S18). The SSLE- and ink-based devices exhibit comparable performance. Notably, the ink-based device shows reduced noise current, resulting in an enhanced D* (Fig. 3c). Furthermore, the ink-based device shows improved stability under a −1 V bias (Fig. S19).

Although comparable single-pixel device performance is obtained, imaging uniformity is equally critical for practical SWIR imaging applications, as it directly governs pixel-to-pixel consistency and system-level image quality. To evaluate array-level performance, the top-illuminated PbS QD photodiodes were monolithically integrated with a custom-designed Complementary Metal-Oxide-Semiconductor (CMOS) readout integrated circuit (ROIC) to form a SWIR image sensor. The spatial resolution was evaluated using the ISO 12233 slanted-edge method, with representative test-chart images. Grayscale values extracted along a representative horizontal pixel area from the captured images to assess imaging uniformity. The SSLE-based imager exhibits a broad and dispersed grayscale distribution across pixels, whereas the ink-based imager maintains a highly uniform grayscale distribution, indicating that the HTL may play a decisive role in governing pixel-to-pixel response (Fig. 3d and e).

Further quantitative analysis supports this observation: the SSLE-based imager shows a Photo photoresponse non-uniformity (PRNU) of 10.57%, with 0.46% hot pixels and 0.21% dead pixels. In contrast, the ink-based imager demonstrates significantly better uniformity, with a PRNU of 5.79%, along with 0.45% hot pixels and only 0.03% dead pixels (Fig. 3f). Consistently, modulation transfer function (MTF) analysis reveals enhanced spatial resolution for the ink-based imager (Fig. S20). In addition, the statistical distribution of noise electrons shows that the ink-based imager has a lower noise-electron level than the SSLE-based imager, indicating reduced readout noise across the imaging array (Fig. S21) [20]. These metrics underscore the notable difference in pixel-level consistency between the two imagers and reinforce the potential decisive influence of the HTL on pixel response uniformity.

### Origins of imaging uniformity

**Figure 4.**
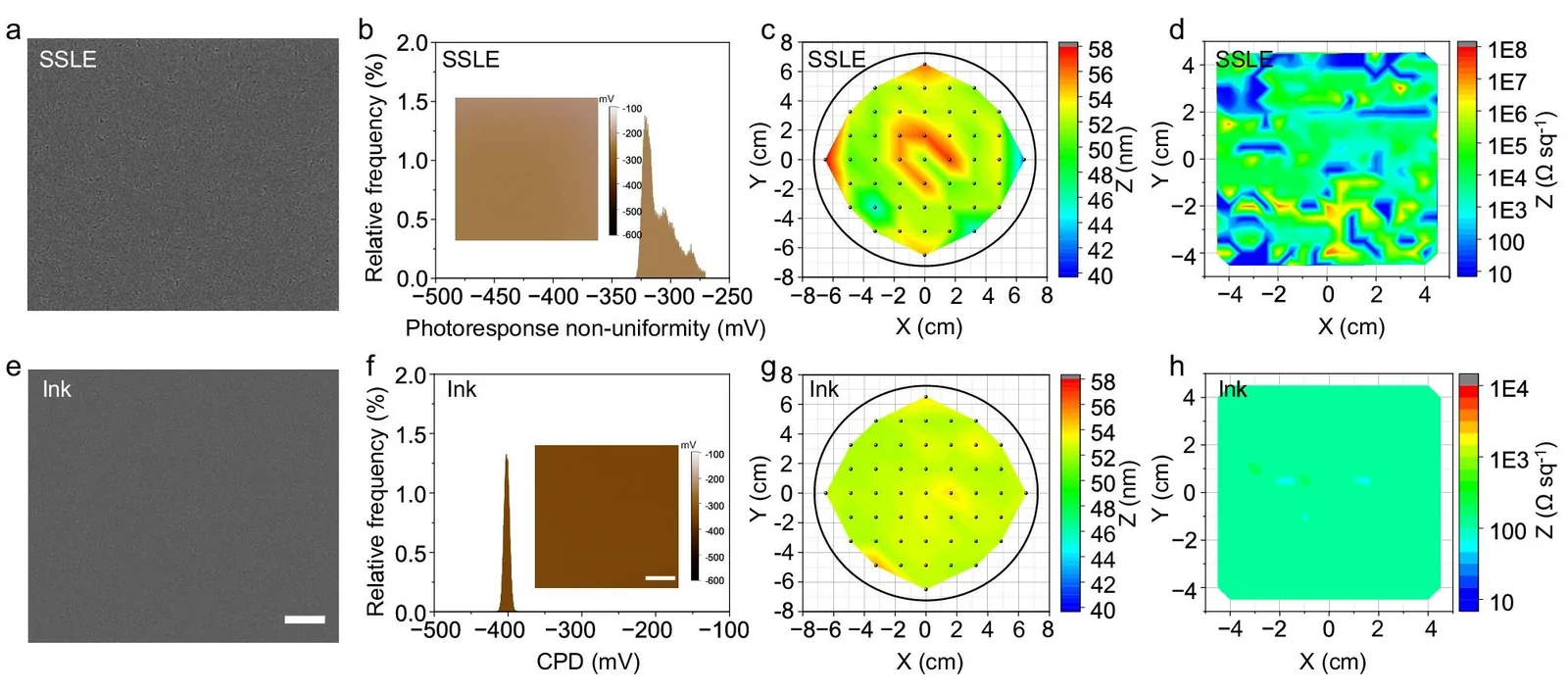
(a, e) The SEM images of SSLE- (a) and ink-based films (e) (the scale bar is 2 μm). (b, f) Analysis of the 2D surface potential distribution based on SSLE- (b) and ink-based films (f); insets: KPFM images (scale bar is 2 μm). (c, g) Ellipsometry thickness mapping of SSLE- (c) and ink-based (g) films on 6-inch Si wafers. (d, h) Sheet-resistance mapping of SSLE- (d) and ink-based films (h) on 10 cm × 10 cm ITO substrate.

To further investigate the origins of the observed differences in pixel-to-pixel uniformity between SSLE- and ink-based devices, we first examined the film morphology using scanning electron microscopy (SEM). The SSLE-processed film exhibits noticeable cracks, which may originate from the inherently disruptive nature of the SSLE process (Fig. 4a and e). In contrast, the ink-processed film maintains a dense and continuous morphology. KPFM was used to further investigate the surface potential and spatial distribution of the HTL film [49]. According to the formula *V_CPD_* = (*φ_sample_ − φ_tip_*), where *φ_tip_* is the work function of the tip, and *φ_sample_* is the sample work function, the electronic structure difference of the HTL films can be obtained by comparing the *V_CPD_*. The ink-based film shows a lower contact potential difference, consistent with stronger p-type character. Notably, the SSLE-based film exhibits a broad surface-potential distribution with a full width at half maximum (FWHM) of approximately 83 mV, which is more than an order of magnitude larger than that of the ink-based film (8 mV) (Fig. 4b and f). This pronounced electronic heterogeneity is fully consistent with the previously observed dispersion in pixel-wise grayscale values, highlighting that both surface-potential uniformity and film morphological uniformity are essential for achieving homogeneous imaging response.

Given the requirements of scalable manufacturing, we next examined and compared the large-area film morphology of SSLE and ink-based films on 6-inch wafers (Fig. S22). Ellipsometry mapping was employed to quantify the film thickness across the wafer. Despite comparable average film thicknesses for the SSLE and ink-based films (52.28 and 52.63 nm, respectively), the SSLE-based film exhibits a substantially larger thickness variation across the wafer (±7.82 nm) compared with the ink-based film (±0.31 nm) (Fig. 4c, g and Fig. S23). Consistently, large-area sheet-resistance mapping of QD films deposited on 10 cm × 10 cm ITO substrates reveals that the ink-based films exhibit a much narrower spatial resistance distribution (151.0 ± 24.1 Ω sq^−1^) and substantially reduced point-to-point variation. In contrast, the SSLE-based film shows highly nonuniform resistance values with large spatial fluctuations extending into the ^M^Ω sq^−1^ regime, confirming that the ink-based process yields a more homogeneous conductive film across the full substrate area (Fig. 4d and h). Taken together, although the SSLE approach can achieve high-performance single-pixel photodetectors, the inherent non-uniformity in film morphology and spatial electronic structure limits its suitability for large-area, scalable manufacturing and uniform imaging. These findings establish the ink-based approach as a practical and scalable materials route toward wafer-level fabrication of low-cost infrared imaging chips.

### Practical SWIR imaging applications

We further demonstrate several application scenarios enabled by our PbS QD imager. The device achieves silicon-penetrating imaging in transmission mode, as expected for SWIR operation (Fig. S24a). In addition, our QD imager can distinguish different solvents—trichloroethylene, ethyl acetate, and water—under 1550 nm illumination (Fig. S24b).

**Figure 5.**
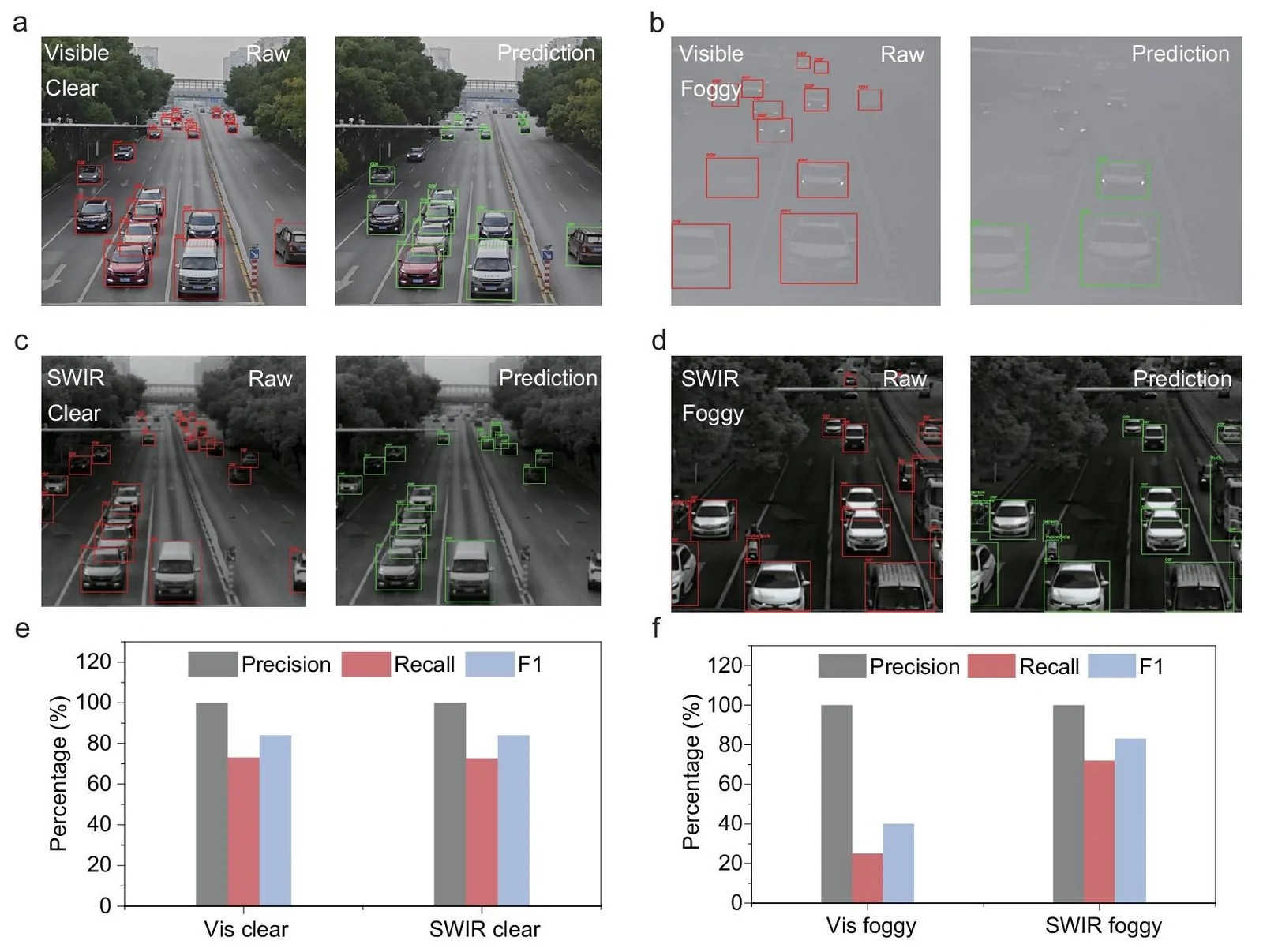
(a, b) Visible-light camera under clear (a) and foggy (b) conditions (left) and corresponding vehicle-recognition results (right). (c, d) QD imager under clear (c) and foggy (d) conditions (left) and corresponding vehicle-recognition results (right). (e, f) Precision, recall, and F1-score of visible and SWIR imaging under clear (e) and foggy conditions (f).

To further evaluate the practical applicability of the SWIR imaging device based on p-type PbS QD inks, the same target detection algorithm with identical parameters was employed to assess the image quality of the QD-based imager in a realistic traffic-scene vehicle-recognition task (Fig. S25). The raw images, manually annotated ground-truth labels, and corresponding detection results were compared (Fig. 5). Under clear-weather conditions, both visible-light and SWIR cameras provide sufficient scene information for reliable vehicle recognition. Vehicles are accurately identified in visible-light images, with detection results closely matching the ground truth (Fig. 5a). A comparable performance is achieved by the SWIR imager under clear conditions (Fig. 5c), where vehicles are clearly resolved and reliably detected, indicating that the PbS QD–based SWIR images retain adequate visual features for computer vision tasks in normal environments. In contrast, the difference between visible-light and SWIR imaging becomes more pronounced under foggy conditions. Strong scattering of visible light by fog degrades image contrast, resulting in blurred vehicle contours and reduced detection performance (Fig. 5b). Consequently, some vehicles are difficult to distinguish, leading to incomplete or unreliable detection results. By comparison, the SWIR imager preserves improved scene visibility under foggy conditions (Fig. 5d), allowing vehicles and smaller objects, such as motorcycles and pedestrians, to remain more discernible. These SWIR images facilitated subsequent object-recognition analysis using the same algorithm, highlighting the benefit of improved image quality under foggy conditions. To further quantitatively evaluate imaging performance, statistical metrics including precision, recall, and F1-score were employed (Fig. 5e and f). Under clear conditions, comparable performance is observed for both visible and SWIR images. In contrast, under foggy conditions, while the precision remains similar, SWIR images exhibit higher recall and F1-score than visible images, indicating improved robustness in reduced-visibility environments.

These results demonstrate that SWIR images acquired using p-type PbS QD inks provide enhanced robustness against adverse weather conditions compared with conventional visible-light imaging. The improved detection performance under fog highlights the potential of the QD-based SWIR imager for all-weather traffic monitoring and related computer vision applications.

## CONCLUSION

In this work, we successfully synthesized p-type PbS QD inks directly and investigated the influence of various thiol-containing small molecules on the growth behavior of QDs during synthesis. By tuning the electron-withdrawing capability of the para-substituents on the thiol ligands, the binding affinity between the ligands and the QD surface could be modulated, thereby controlling the p-type electronic characteristics. Notably, the first-excitonic absorption peak of the resulting QDs can be extended from 1100 nm to beyond 2000 nm, significantly broader than the <1700 nm first-excitonic absorption peak of previously reported p-type QD inks prepared via SPLE. The p-type QD ink method eliminates the need for ligand exchange during the fabrication of conductive films, thereby exhibiting dense, crack-free morphology and spatially uniform electronic structure. Moreover, the films maintain stable morphology, suitable energy-level alignment, and surface-potential distribution. Notably, the ink-derived films maintain excellent morphological uniformity even on 6-inch silicon substrates. The superior film uniformity further enables uniform pixel performance in imaging applications. SWIR imagers based on the p-type PbS QD inks demonstrate uniform grayscale response and robust imaging performance under both clear and heavy-fog conditions. When combined with the object-recognition algorithm, the system enables reliable vehicle identification. This synthetic strategy provides a scalable and stable materials platform for uniform large-area PbS QD films, supporting future wafer-level SWIR optoelectronic applications.

## METHODS

### Direct synthesis of p-type PbS QD inks

Pb(SCN)_2_ (6 mmol) and thiol ligands (1 mmol) were dissolved in DMF (9 mL) and stirred at room temperature. A mixture of BA (1 mL) and diphenylthiourea (1 mmol) was then rapidly injected. After 5 min, the reaction mixture was transferred to a nitrogen atmosphere and mixed with toluene (10 mL). Following centrifugation at 8000 rpm, the supernatant was collected and subjected to a second toluene precipitation step (10 mL) under the same conditions to obtain the p-type PbS QD solid. Synthesis procedures for p-type conductive PbS QDs of different sizes are described in the Supporting Information.

### Single-pixel photodiode fabrication

An 80 nm Au bottom electrode was deposited by electron-beam evaporation and patterned by photolithography and lift-off to define a 600 × 600 μm photosensitive area, followed by a 40 nm NiO_x_ hole-transport layer deposited by RF sputtering. The hole-extraction layer comprised either p-type PbS QD inks (950 nm first-excitonic peak) or two EDT-treated PbS QD layers (880 nm; 0.01 vol% EDT in acetonitrile). The active QD layer was spin-coated as described previously [29]. A 15 nm C_60_ layer, 25 nm SnO_2_ layer, and 200 nm ITO top electrode were subsequently deposited by thermal evaporation, atomic layer deposition, and room-temperature DC sputtering, respectively.

### QD image sensor fabrication

A 640 × 512 CMOS ROIC (13.5 μm pitch; 8.64 mm × 6.91 mm active area) was integrated with the same PbS QD device stack used for the single-pixel photodiodes. After SnO_2_ deposition, the device stack was photolithographically patterned using AZ5214 photoresist and etched by ICP with Ar/Cl_2_. The top ITO electrode and Al_2_O_3_ encapsulation layer were then deposited, patterned with AZ5214, and etched in 0.7% HCl for 3 min. Finally, the QD image sensor was packaged in a PGA-format metal casing to provide hermetic sealing and electrical interconnection with the camera module.

### Image capture and characterizations

The noise voltage ${{\overline{V}}_{\mathrm{N}}}$ is defined as the root mean square noise voltage across all pixels. The noise voltage for (*i, j*) pixel *V*_N_(*i, j*) is calculated as:


\begin{eqnarray*}
{V}_{\mathrm{N}}(i{\mathrm{,}}j) &=& \frac{1}{K}\sqrt {\frac{1}{{F - 1}}\sum\limits_{f = 1}^F {\left\{ {\overline {{V}_{{\mathrm{DS}}}} \left[ {(i{\mathrm{,}}j),{T}_0} \right] - {V}_{DS}{{\left[ {(i{\mathrm{,}}j),{T}_0,f} \right]}}^2} \right\}}}\\
{{\overline V }_{\mathrm{N}}} &=& \frac{1}{{M \times N}}\sum\limits_{i = 1}^M {\sum\limits_{j = 1}^N {{{V}_N}(i,j)}},
\end{eqnarray*}

where (*K* = 1) is the system gain, (*F* = 100) is the number of acquired frames, *T*_0_ is the test temperature, and (*M* = 640) and (*N* = 512) are the numbers of columns and rows in the image sensor, respectively.

PRNU was calculated as the standard deviation of pixel responsivities normalized to the average responsivity:


\begin{eqnarray*}
&&{\mathrm{PRNU}} =\nonumber\\
&&\quad \frac{1}{{\overline R }}\sqrt {\frac{1}{{M \times N}}\sum\limits_{i = {\mathrm{1}}}^M {\sum\limits_{j = 1}^N {{{{\left[ {R(i,j) - \bar R } \right]}}^2}} } } \times 100\% .
\end{eqnarray*}

The average responsivity $\bar{R}$ was obtained from all pixels, with *R*(*i, j*) denoting the responsivity of pixel (*i, j*).


\begin{eqnarray*}
R(i,j) = \frac{{{{V}_s}(i,j)}}{P},
\end{eqnarray*}

\begin{eqnarray*}
\bar R = \frac{1}{{M \times N}}\sum\limits_{i = 1}^M {\sum\limits_{j = 1}^N {R(i,j)} } ,
\end{eqnarray*}

where *V*_S_(*i, j*) represents the response voltage of each pixel, *P* is the blackbody output power.

The dead pixel is defined as the pixel with *R*(*i, j*)< $\frac{1}{2}\bar{R}$, whereas hot pixels were defined as pixels with *V*_N_(*i, j*) > $2{{\overline{V}}_N}$. Their respective ratios were calculated relative to the total pixel number. The effective pixel ratio (*N*_ef_) is defined as:


\begin{eqnarray*}
{{N}_{{\mathrm{ef}}}} = (1 - {{N}_{{\mathrm{dead}}}} - N{}_{{\mathrm{hot}}}) \times 100\% ,
\end{eqnarray*}

where *N_dead_* and *N_hot_* are the fractions of dead and hot pixels, respectively.

Spatial resolution was evaluated using the ISO 12233 slanted-edge method. The normalized MTF was obtained from the Fourier transform of the line-spread function, and MTF50 was defined as the spatial frequency at 50% MTF.

## Supplementary Material

nwag421_Supplemental_File
