# Supplementary material for: Direct synthesis of p-type PbS quantum dot inks toward wafer-scale SWIR imaging
Source: Natl Sci Rev. 2026 Jul 11;13(16):nwag421. doi: 10.1093/nsr/nwag421 (PMC13525715; doi:10.1093/nsr/nwag421)
Supplement: Supplementary file 1 [file nwag421_supplemental_file.pdf]

Supporting Information

Direct Synthesis of p-Type PbS Quantum Dot Inks toward Wafer-Scale SWIR Imaging

Yang Liu[1,#], Shengyu Ma[1,#], Chengjie Deng[2,#], Chunmeng Liu[3,#], Yu Yin[1], Kunyuan Lu[1], Guozheng Shi[1], Lin Yuan[1], Yao Yin[1], Juncheng Zhu[1], Xinyang Huang[1], Lizhen Huang[1], Jianlong Xu[1,*], Liang Gao[2,*], Zeke Liu[1,*] and Wanli Ma[1,*]

[1]State Key Laboratory of Bioinspired Interfacial Materials Science, Institute of Functional Nano & Soft Materials (FUNSOM), Soochow University, Suzhou 215123, China;

[2]Wuhan National Laboratory for Optoelectronics (WNLO) and School of Optical and Electronic Information, Huazhong University of Science and Technology, Wuhan 430070, China;

[3]Division of Physical Science and Engineering, Center for Renewable Energy and Storage Technologies (CREST), King Abdullah University of Science and Technology, Thuwal 23955-6900, Saudi Arabia

***Corresponding authors**. E-mails: xujianlong@suda.edu.cn; highlight@hust.edu.cn; zkliu@suda.edu.cn; wlma@suda.edu.cn

[#]Equally contributed to this work

Methods in the supplementary file

Materials:

Lead iodide (PbI_2, 99+%, Sigma Aldrich), Lead acetate trihydrate ($PbAc_2$ $3H_2O$, 99%, Alfa), Lead thiocyanate ($Pb(SCN)_2$, 99+%, Sigma Aldrich), Hexamethyldisilathiane (TMS-S, 98%, J&K), Diphenylthiourea (DPhTA, 99%, Sigma Aldrich), Toluene (95%, Qiangsheng), N, N'-dimethylformamide (DMF, 99.8% anhydrous, J&K), Butylamine (BA, >99%, TCI), Acetonitrile (ACN, 99%, J&K), 4-Methoxybenzenethiol (99%, Leyan), 4-Aminobenzenethiol (99%, J&K), 4-Chlorothiophenol (99%, J&K), 4-Mercaptobenzonitrile (99%, Sigma Aldrich), and 4-Nitrothiophenol (99%, Sigma Aldrich).

Synthesis of Size-tunable p-type PbS QDs

6 mmol of $Pb(SCN)_2$ and 1 mmol of thiol ligands were dissolved in the 9 mL DMF. The mixture was stirred at 70°C until the solid was completely dissolved. Next, 0.25 mmol of diphenylthiourea was dissolved in 500 μL of butylamine as the sulfur precursor for seed synthesis. Then, 2.5 mmol of diphenylthiourea was dissolved in a DMF-butylamine solution with a volume ratio of 4:1, at a concentration of 0.5-2 M, and used as the sulfur source for the subsequent growth of PbS QDs. The sulfur source for seed synthesis was rapidly injected into the lead precursor solution to initiate the reaction, causing the solution to immediately turn black. After 2 minutes of reaction at 70°C, 250 μL of the sulfur source for subsequent growth was added to the reaction mixture every minute. The reaction solution was transferred to a nitrogen-protected environment, and 10 mL of toluene was added, followed by shaking. The mixture was centrifuged at 8000 rpm, and the supernatant was collected. Additional toluene was added to the supernatant, followed by shaking and centrifugation at 8000 rpm to obtain the p-type PbS QD solid. The amount of toluene depends on the QD size. The resulting PbS QD solids were stored in a nitrogen-protected environment.

DFT Method

The core-valence electron interaction was represented by the project-augmented wave

(PAW) method [1]. Dispersion interactions were taken into account by the DFT-D3 method [2]. The valence electrons were expanded in a plane-wave basis set with a cutoff energy of 450 eV. The geometry optimization is stopped until the maximal force on each relaxed atom is less than 0.05 eV $Å^{-1}$. The (111) surface of PbS was selected to construct a model with the Device Studio software [3]. PbS(111) surface was modeled by a four-layer slab model with a vacuum of 20 Å, which corresponds to a k-points mesh of 1×1×1. During the structural optimization, the bottom two layers were fixed, and the top two layers and the adsorbates on the surface were completely relaxed. The adsorption energy (E_{ads}) of the molecule was calculated as: $E_{ads} = E_{x/surf} - E_{surf} - E_{x}$, where $E_{x/surf}$, E_{surf} and E_{x} are the energy of the surface with adsorbate, the clean surface and the gaseous molecule, respectively. The more negative E_{ads} represents the stronger the adsorption strength.

Measurement

UV-vis NIR spectra were recorded on a Perkin Elmer model Lambda 750. XRD patterns were obtained on a desktop diffractometer (D2 PHASER, Bruker) with a Cu Kα source (λ = 1.54056 Å). The PbS QD films for the UPS/XPS test were made by spin-coating ~200 nm thick film onto a silicon substrate. PL spectra of QD solution were obtained on FluoroMax-4 spectrofluorometer (HORIBA Scientific) with excitation at 750 nm. Transmission electron microscopy (TEM), scanning electron microscopy (SEM) and elemental mapping were determined by a FEI Talos F200X (Thermo Fisher) transmission electron microscope. The KPFM measurements were performed in Peak Force KPFM (PF-KPFM) mode using silicon cantilevers (spring constant ~40 N m^{-1}). The scan area for each measurement was set to 5 × 5 μm^2. Prior to measurement, the tip work function was calibrated using a reference sample. The sheet resistances of QD films on 10 ×10 cm ITO were collected by CDE ResMap 178 fourprobe resistance tester. The uniformity of PbS film on a 6 in. silicon wafer was measured by ellipsometry RC2. The current-voltage characteristics in dark conditions were measured by a semiconductor parameter analyzer (Keysight B1500A) connected to a high-vacuum (<

10^{-3} Pa) probe station (Cindbest, CGO-4) at room temperature.

Image Capture and Characterization

According to the GB/T 17444-2013 standard, average responsivity, effective pixel ratio, and photo-response non-uniformity (PRNU) of the device were evaluated using a blackbody source held at 873 K with an aperture diameter of 5 cm. The blackbody source was positioned 60 cm from the image sensor during measurement. A set of 100 frames was collected and used as the basis for parameter extraction.

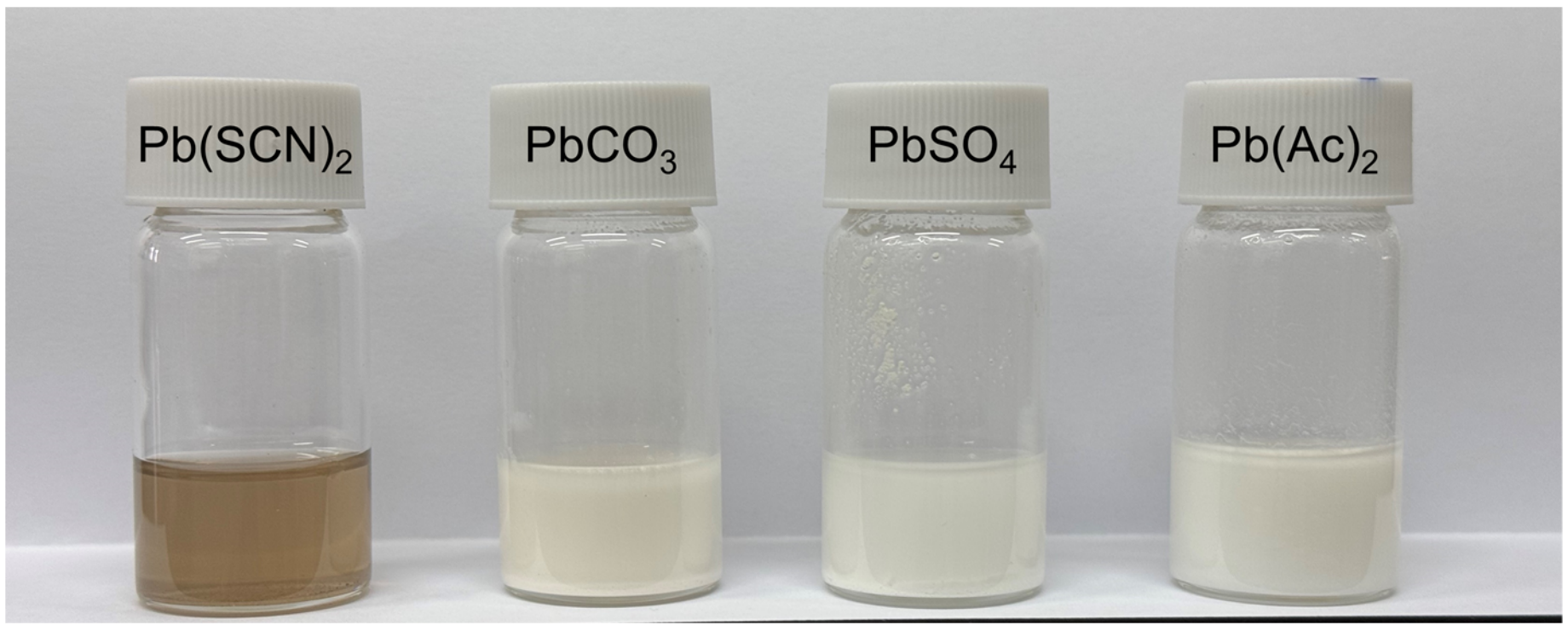

Figure S1. The image of different Pb salts dissolved in DMF.

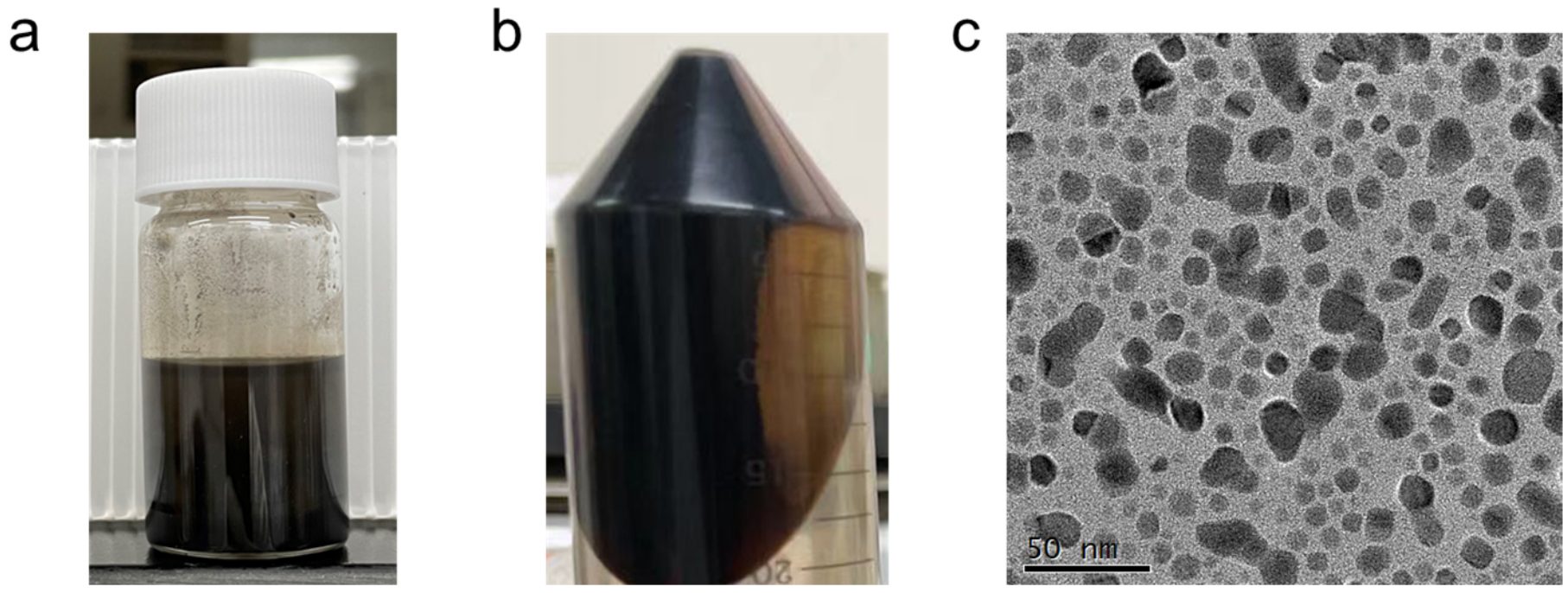

Figure S2. (a) The image of the reaction without small-molecule ligand. (b) Photograph of the product after centrifugation. (c) The TEM image of PbS without small-molecule ligand.

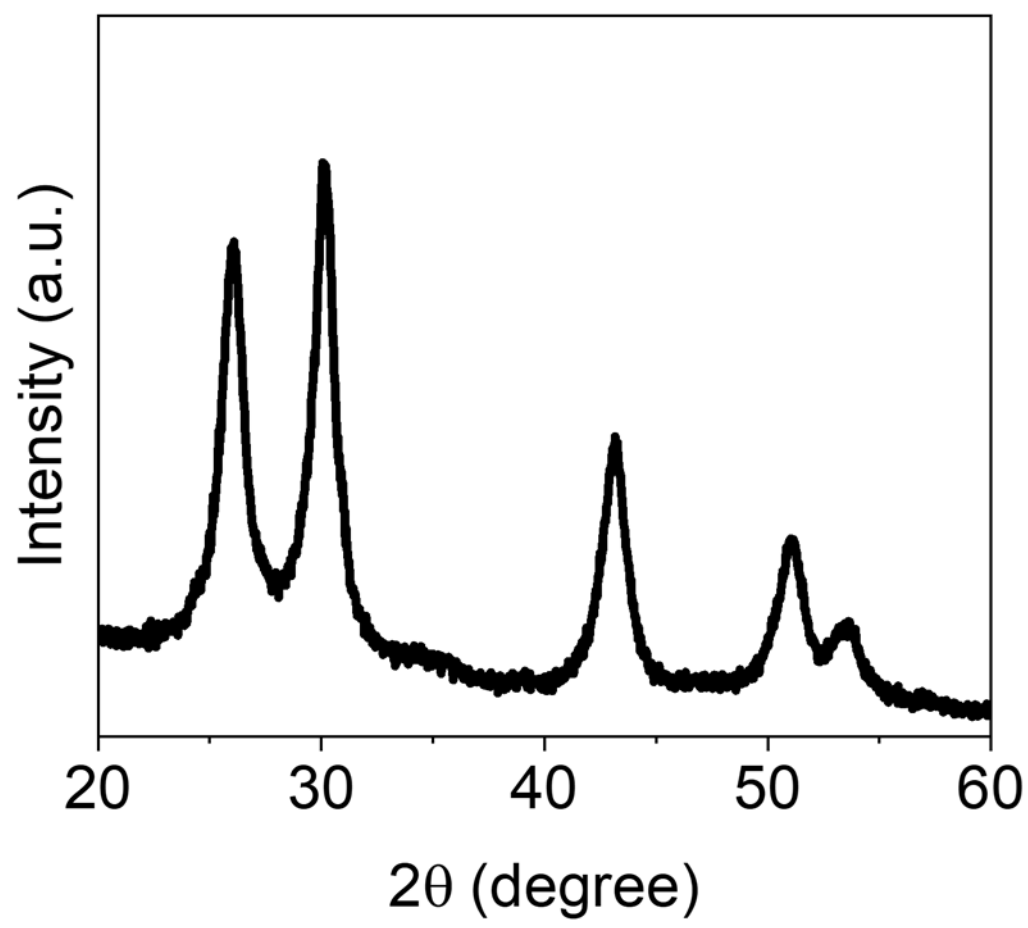

Figure S3. XRD pattern of the product synthesized without introducing small thiol ligand.

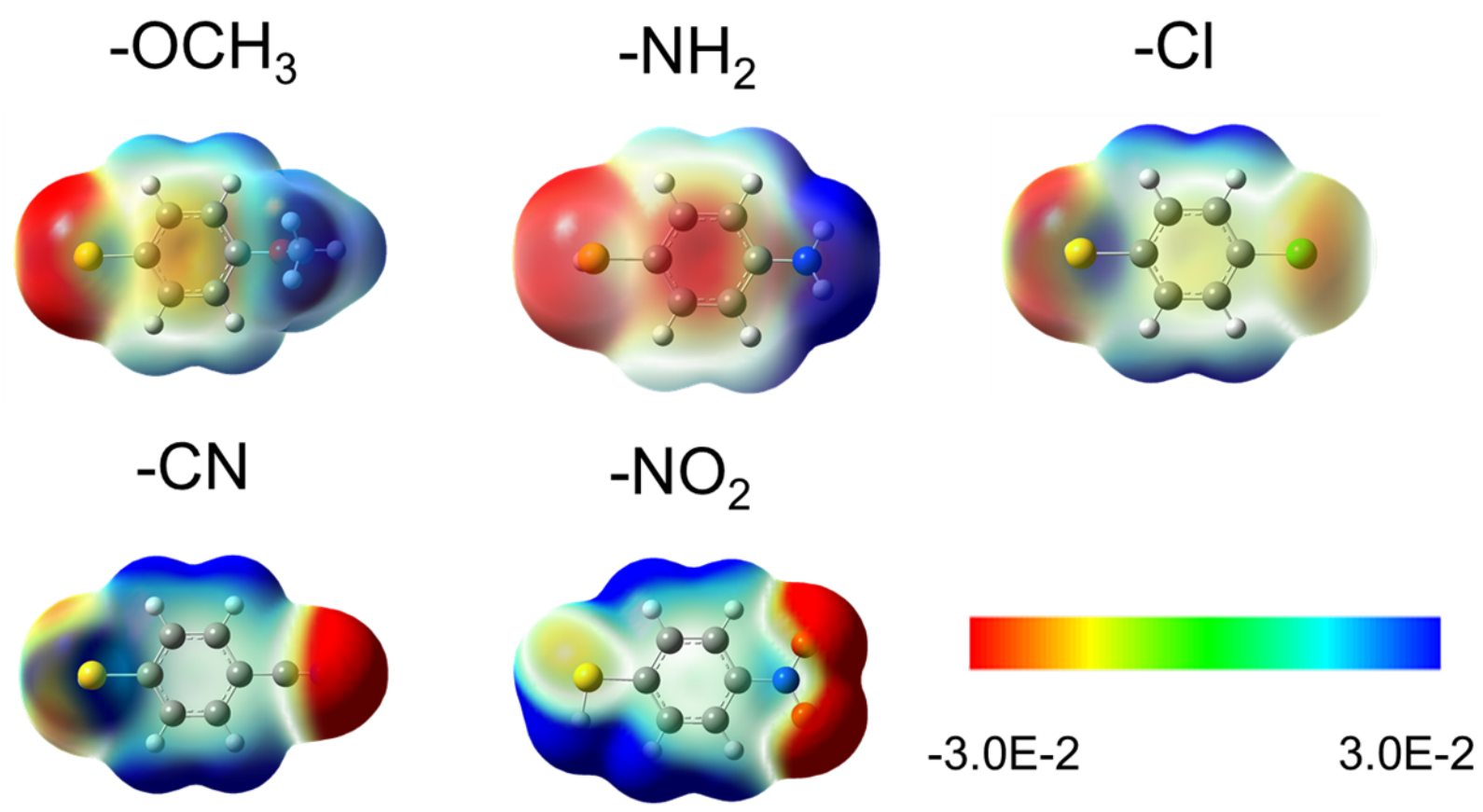

Figure S4. The electrostatic potential (ESP) mapping of different molecules.

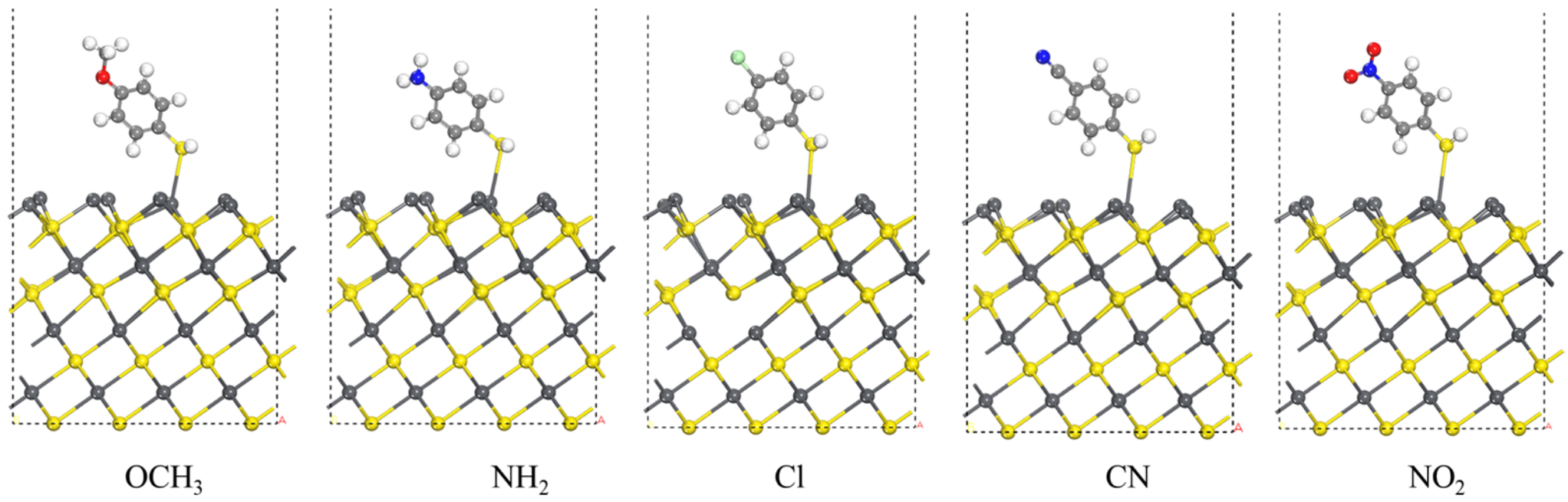

Figure S5. Adsorption structures of molecules with different substituent groups and the corresponding adsorption energy.

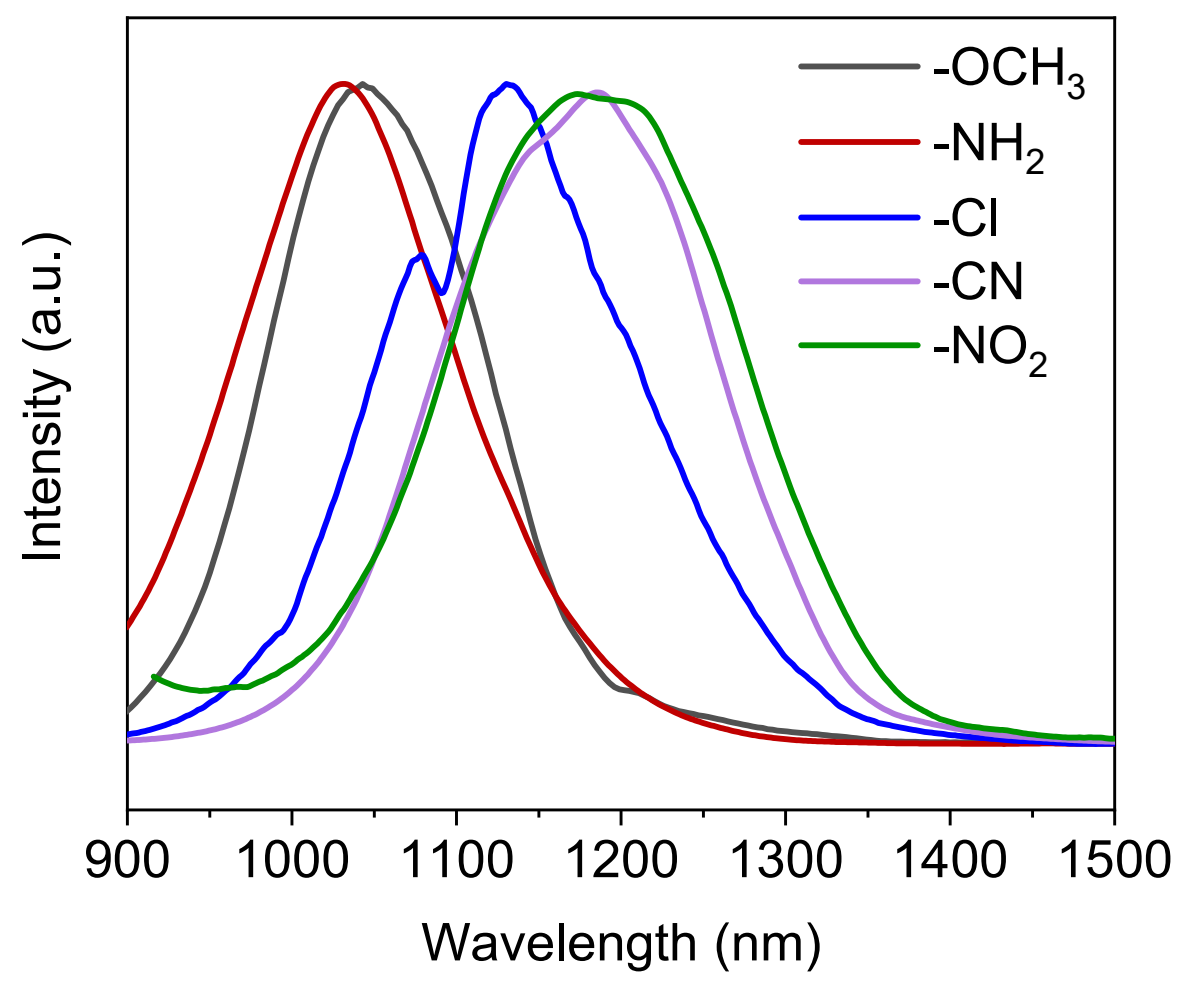

Figure S6. PL spectra of PbS QDs synthesized with different molecules.

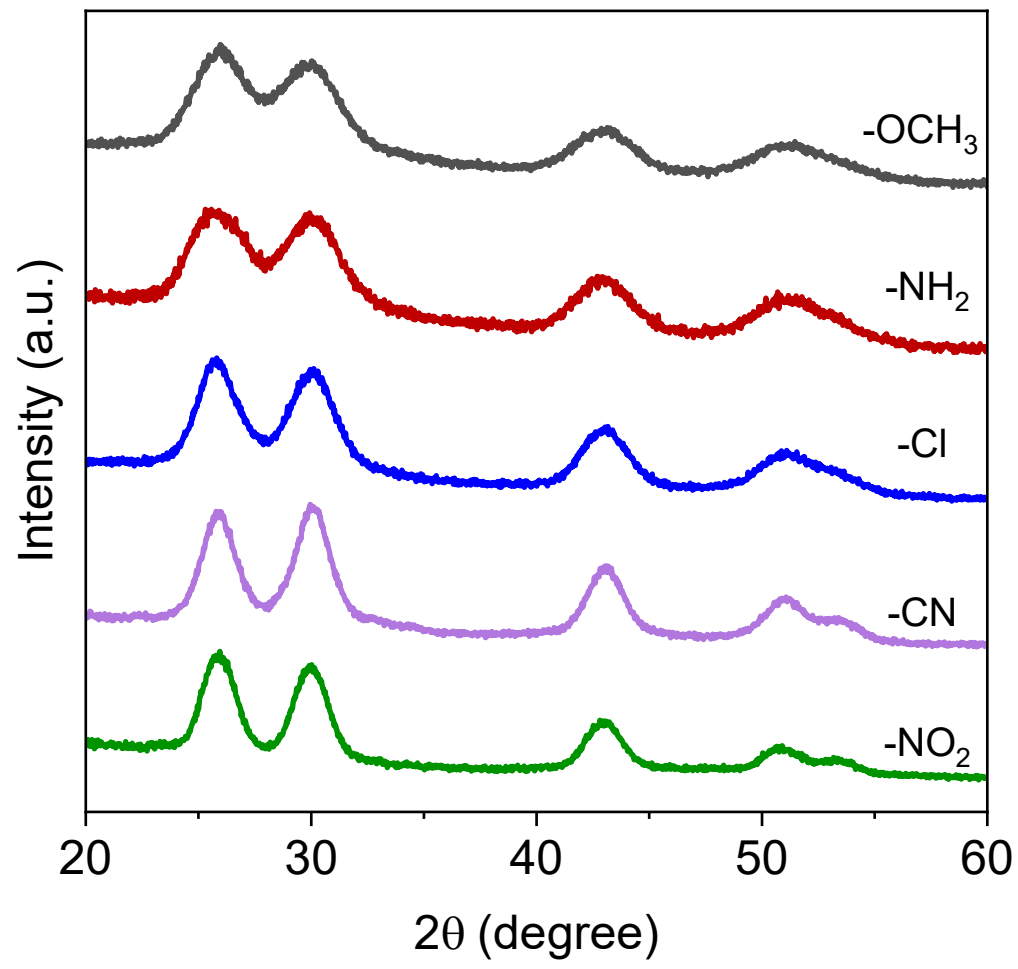

Figure S7. XRD patterns of PbS QDs synthesized with different molecules.

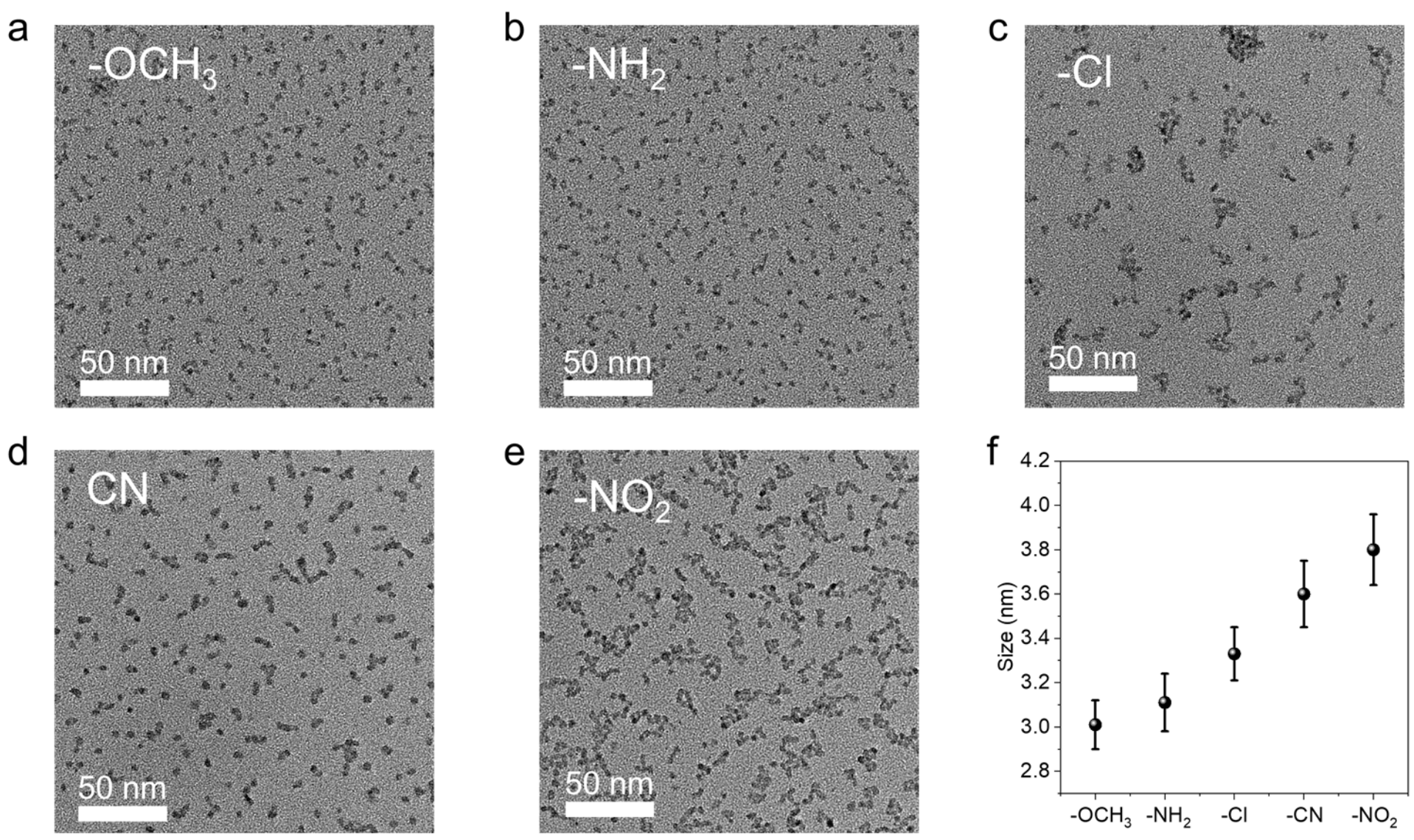

Figure S8. (a-e) TEM images of PbS QDs synthesized with different small molecules. (f) Size statistics.

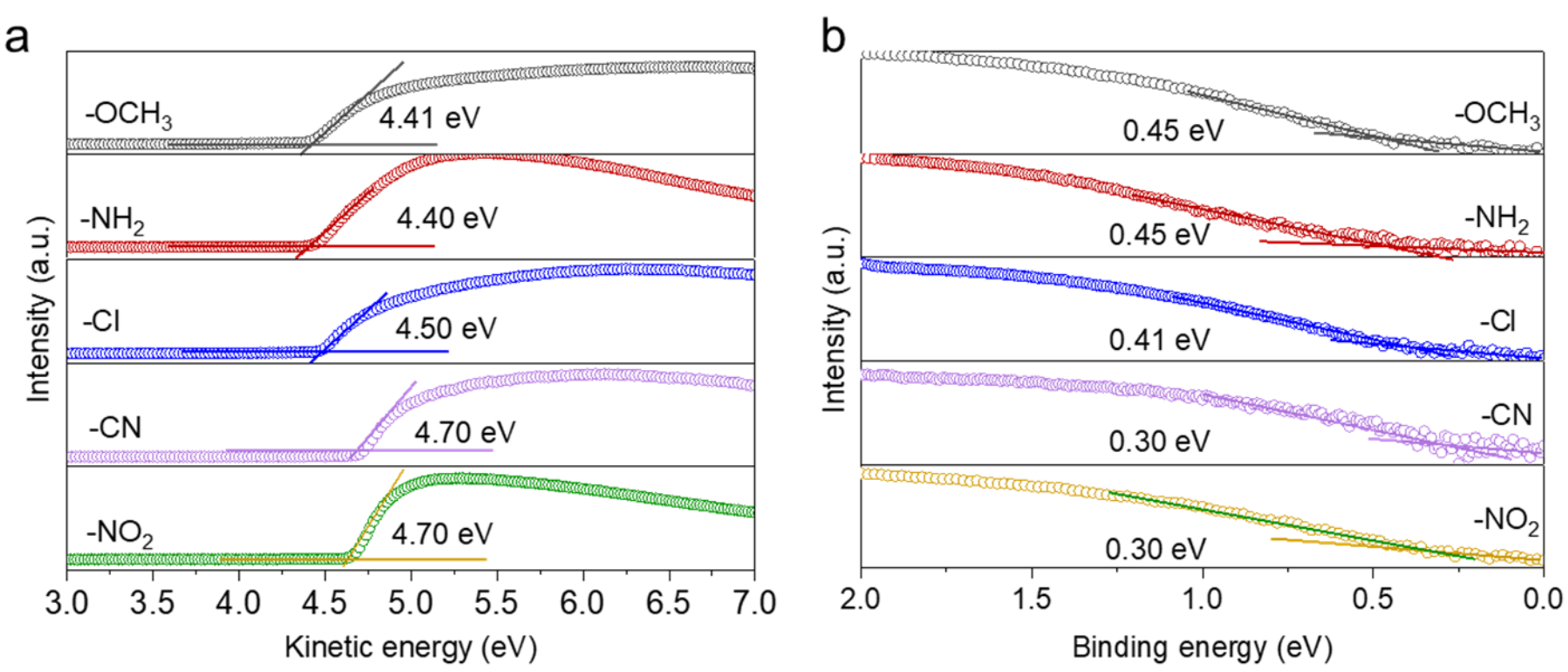

Figure S9. (a, b) UPS spectra of p-type QDs with different small molecules.

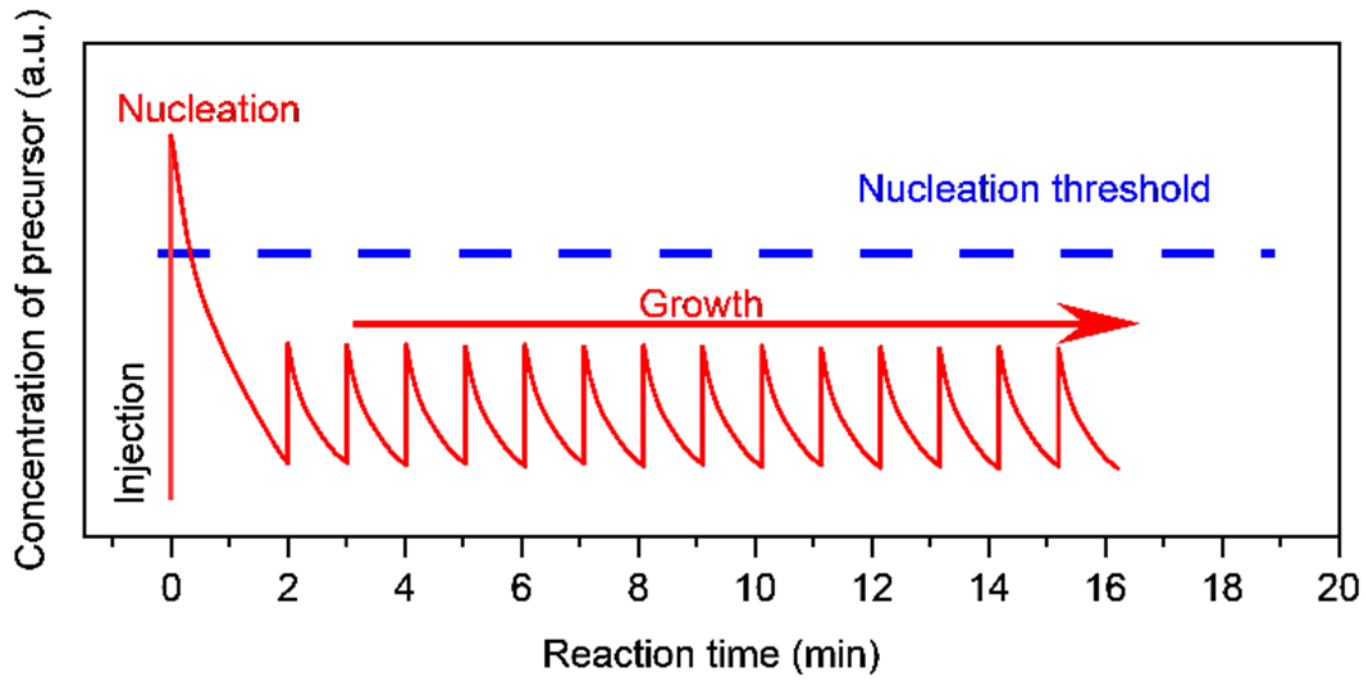

Figure S10. Illustration of the two-step synthesis process. The first step is the formation of initial seed crystals from the first injection of sulfur precursors. The second step is the multiple injections of small doses of sulfur precursors for further growth.

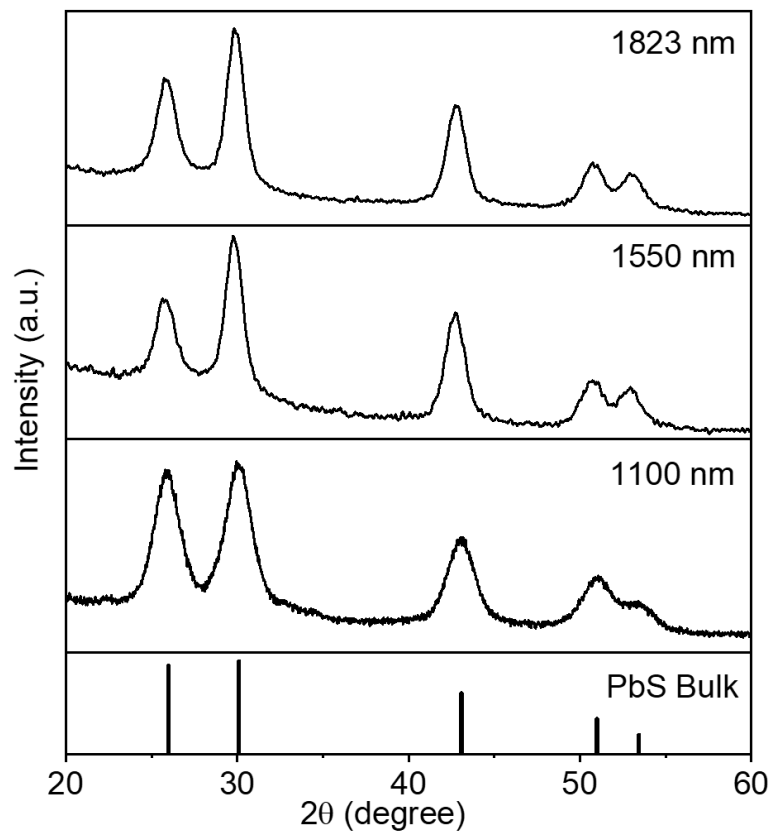

Figure S11. XRD patterns of PbS QDs with different sizes (The numbers in the figure denote the absorption peak positions of the QDs).

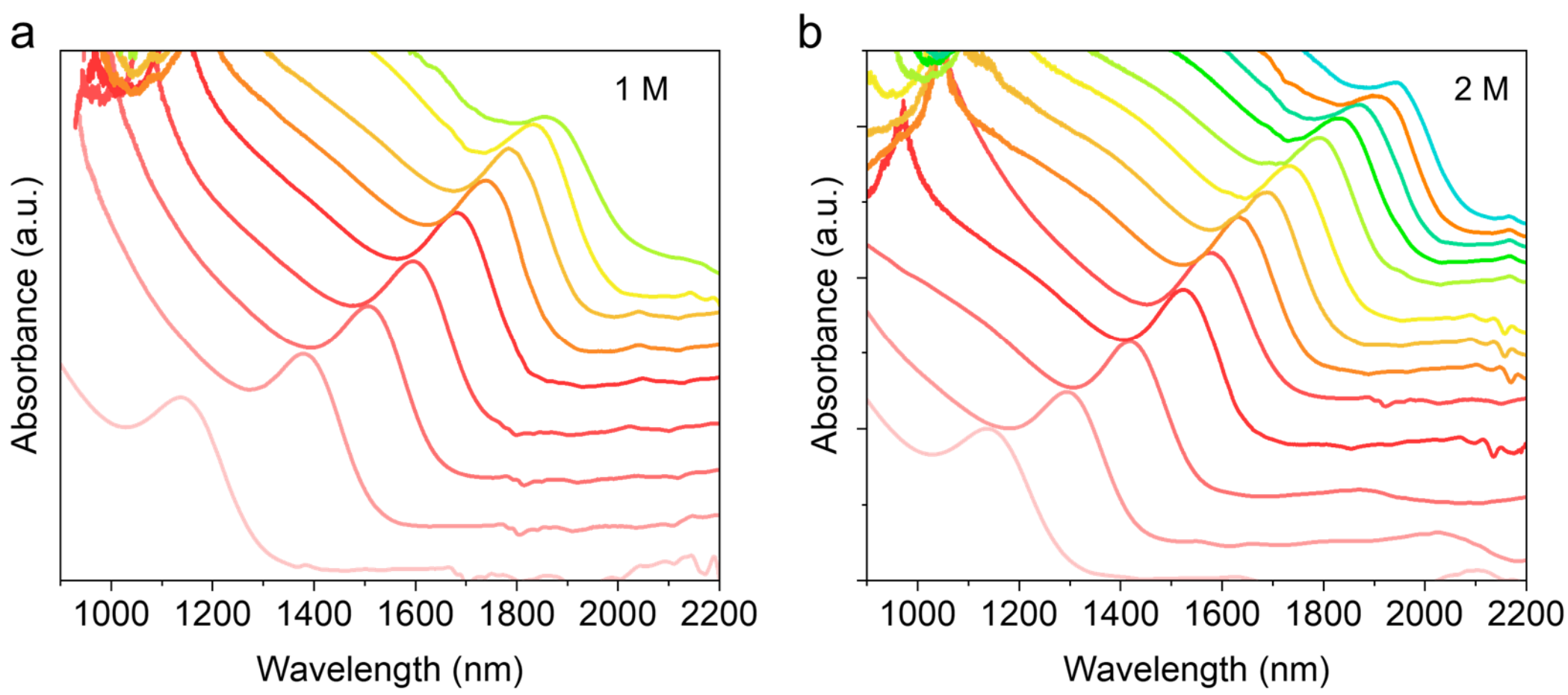

Figure S12. Absorbance spectra of PbS QDs obtained by injecting the concentration of S precursors in the second step (a) 1 M, (b) 2 M.

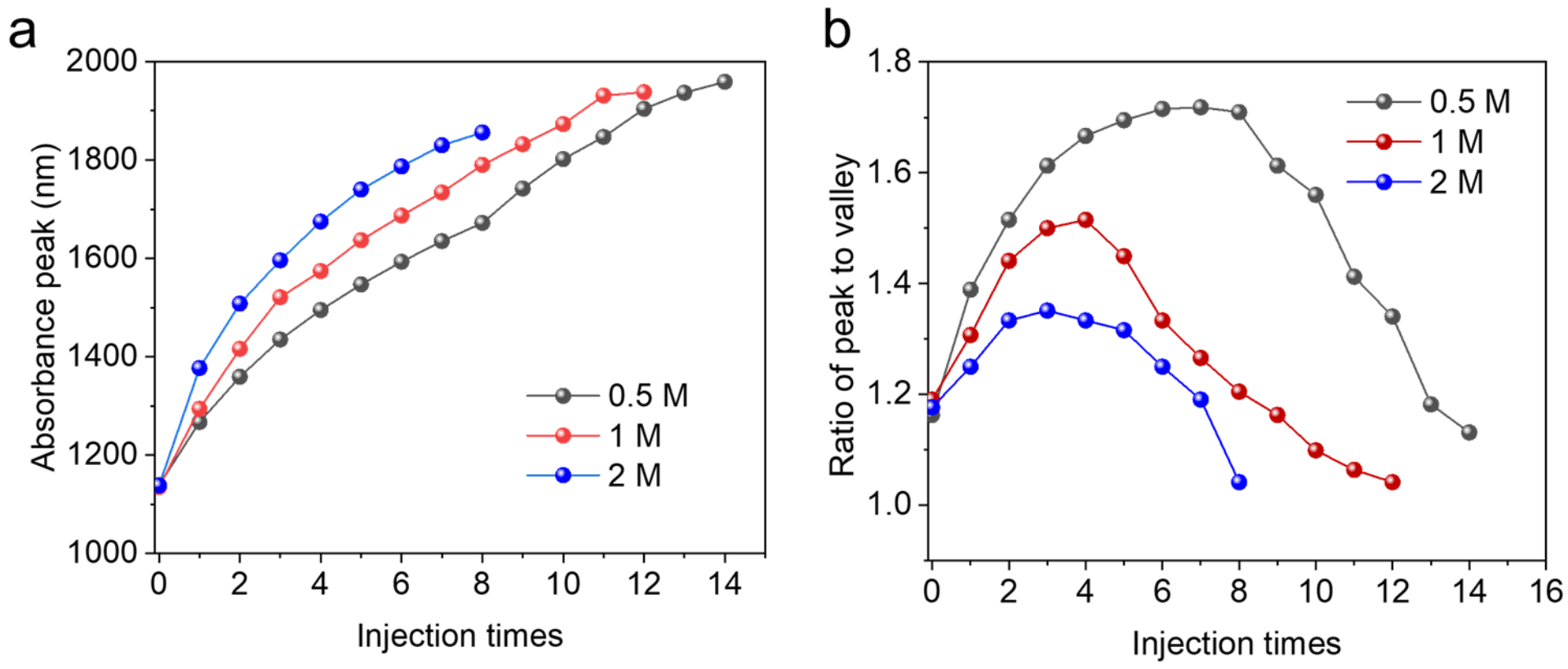

Figure S13. (a) Absorption peak position, (b) peak to valley ratio of PbS QDs obtained by injecting the concentration of S precursor in the second step.

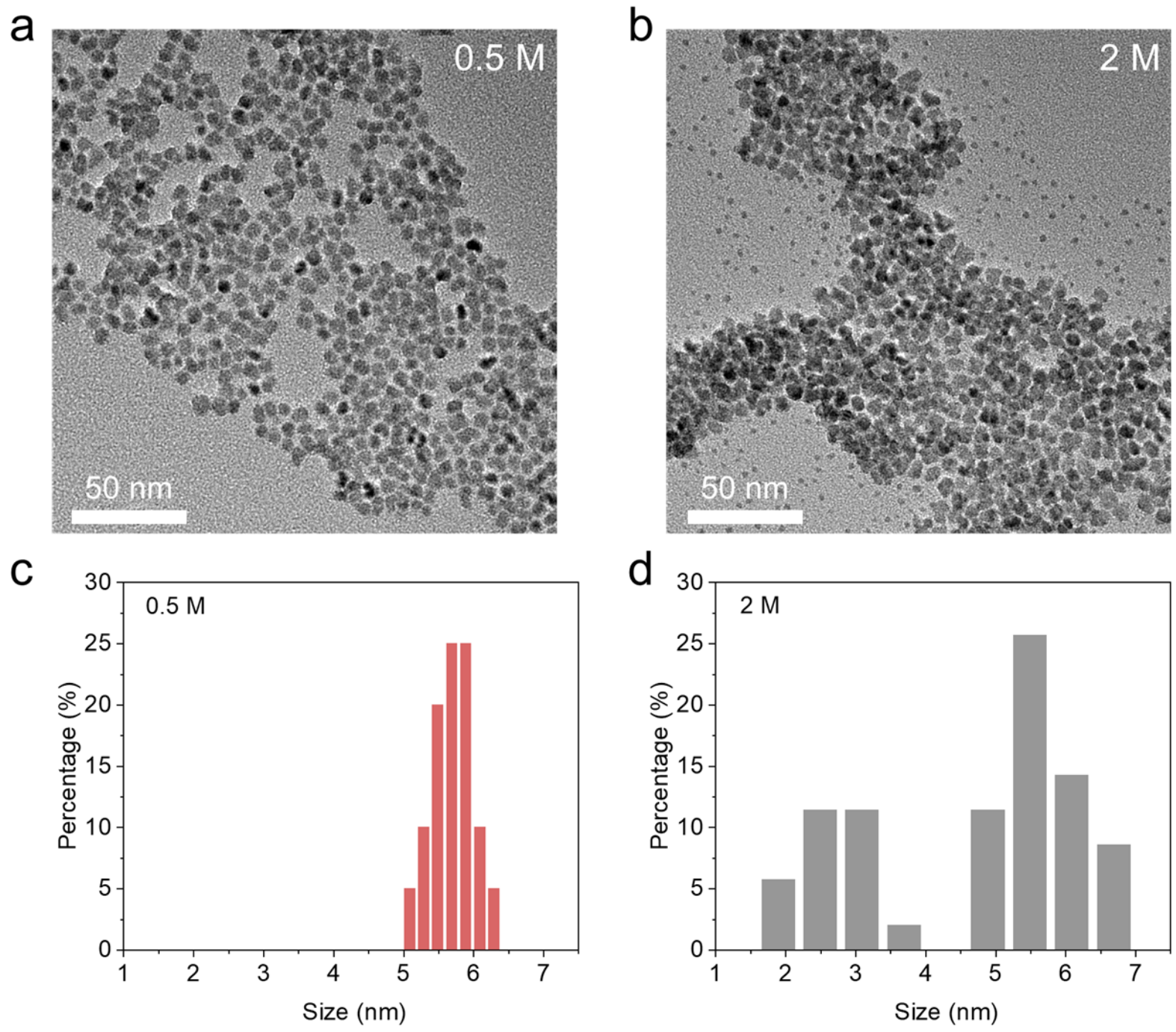

Figure S14. (a,b) TEM images of QDs synthesized with sulfur precursor concentrations of 0.5 M (a) and 2 M (b); (c, d) Size distributions of QDs synthesized with 0.5 M (c) and 2 M (d) sulfur precursor concentrations.

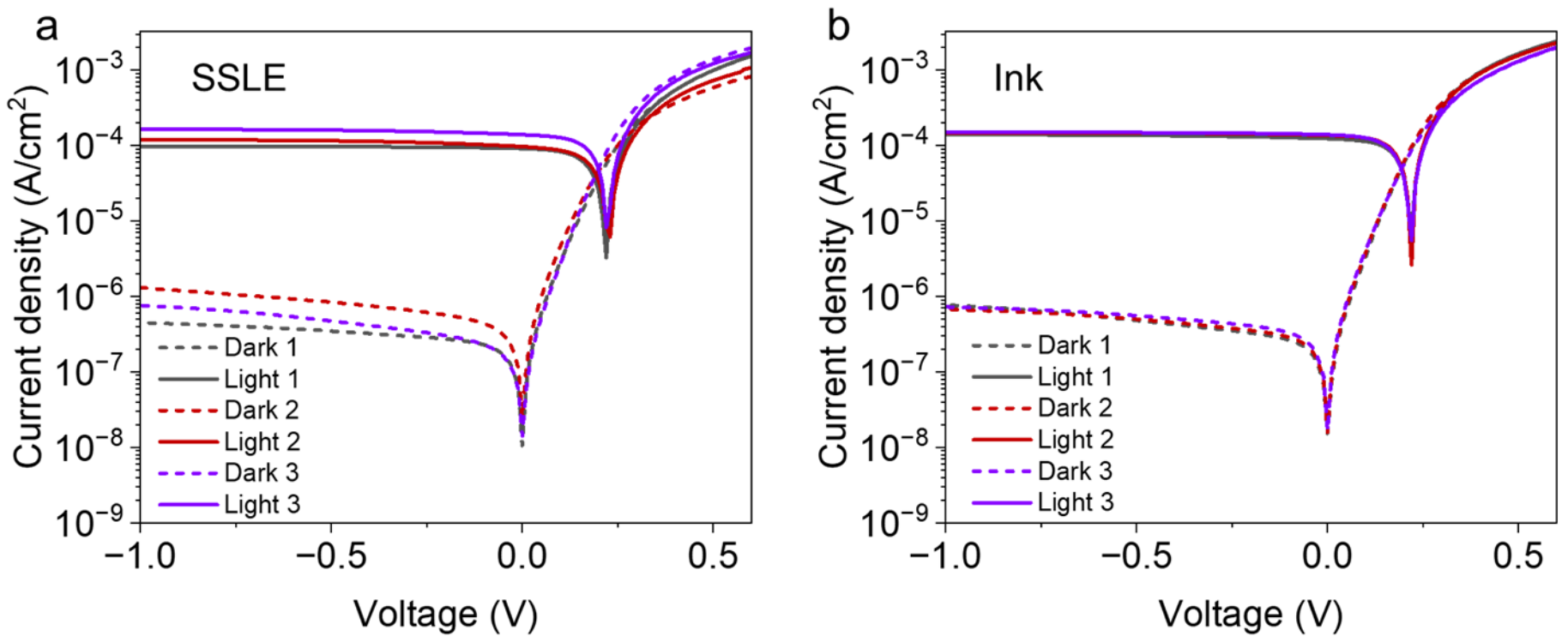

Figure S15. (a, b) Batch-to-batch reproducibility of SSLE- and ink-based devices measured under 1550 nm illumination at an intensity of 250 mW cm^{-2}.

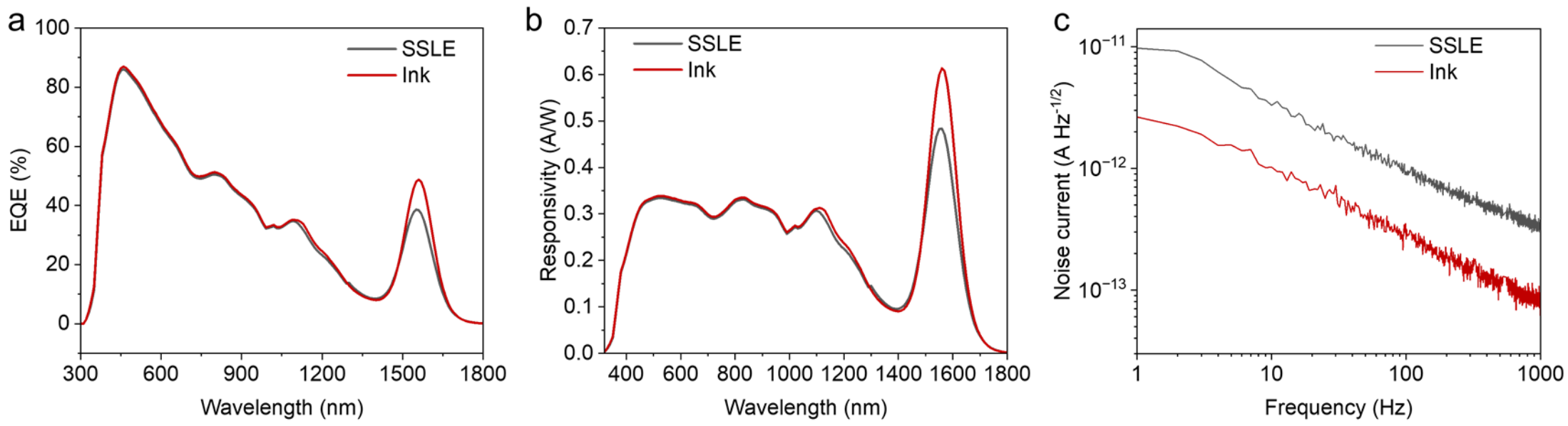

Figure S16. (a-c) EQE (a), responsivity (b), noise current spectrum (c) of the SSLE- and ink-based devices.

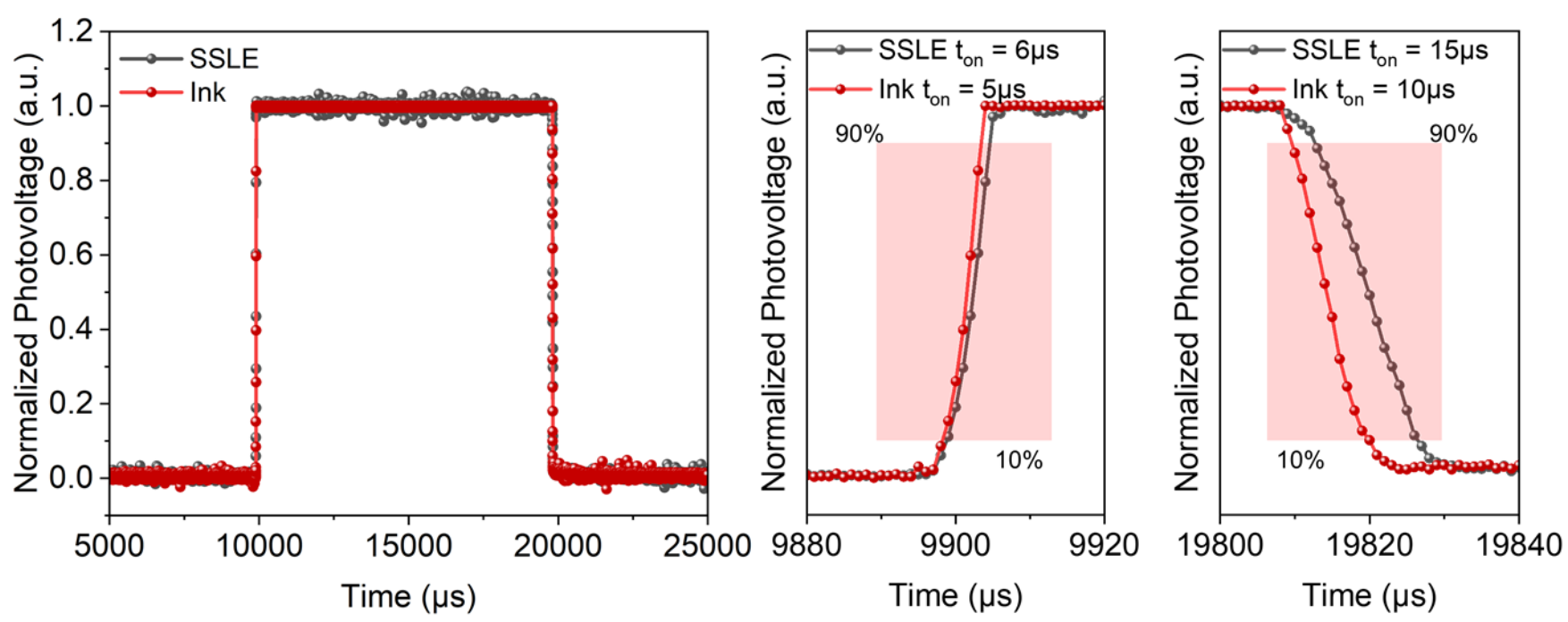

Figure S17. Response time of the SSLE- and ink-based devices.

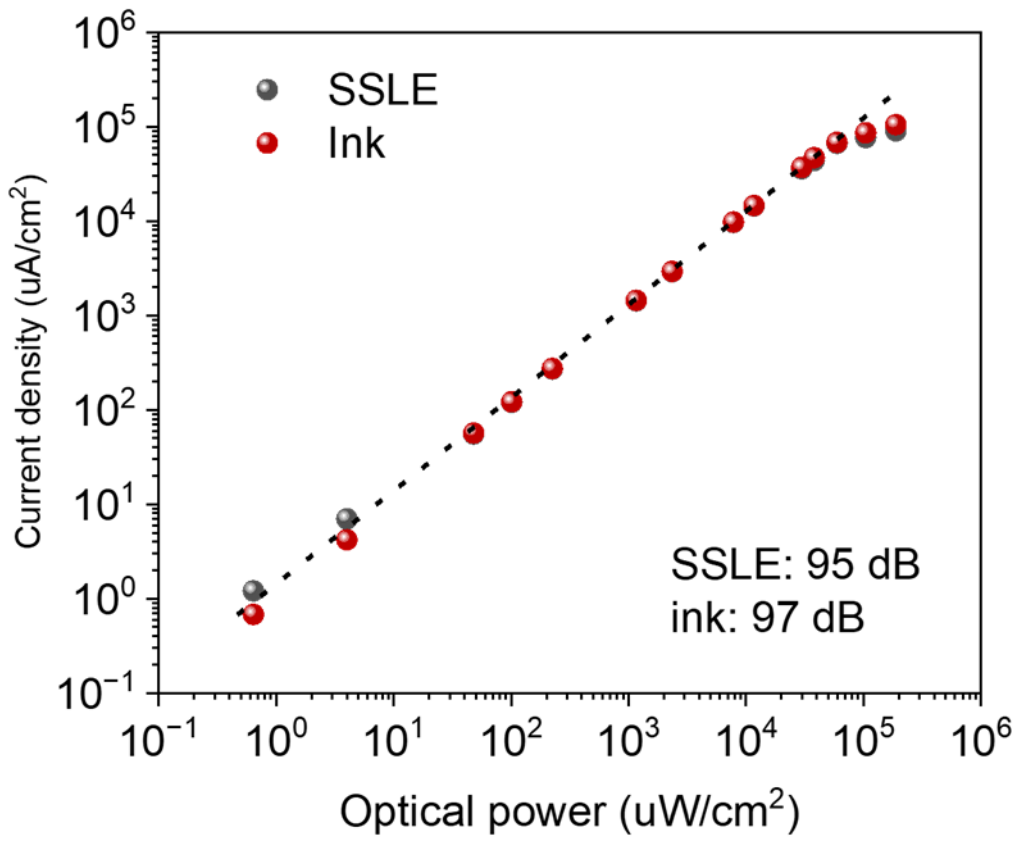

Figure S18. Linear dynamic range (LDR) of the SSLE- and ink-based devices.

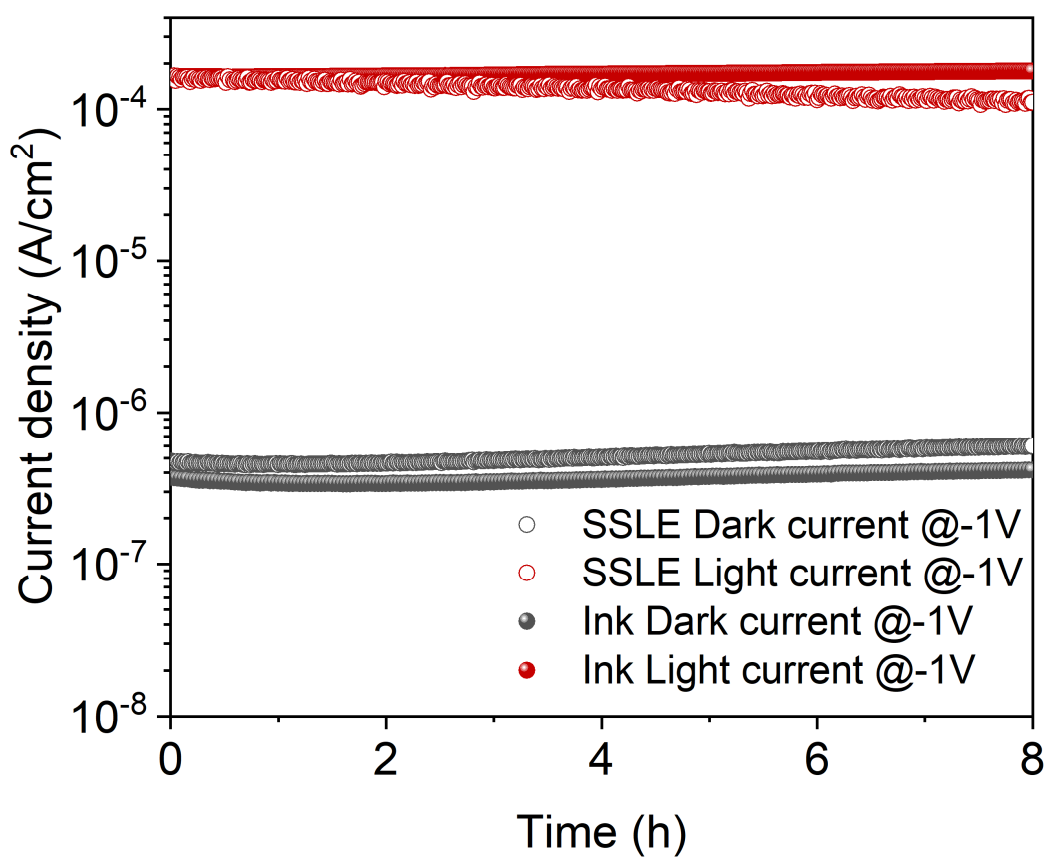

Figure S19. The operational stability of the SSLE- and ink-based devices under 1550 nm illumination (light intensity is 250 mW cm^{-2}).

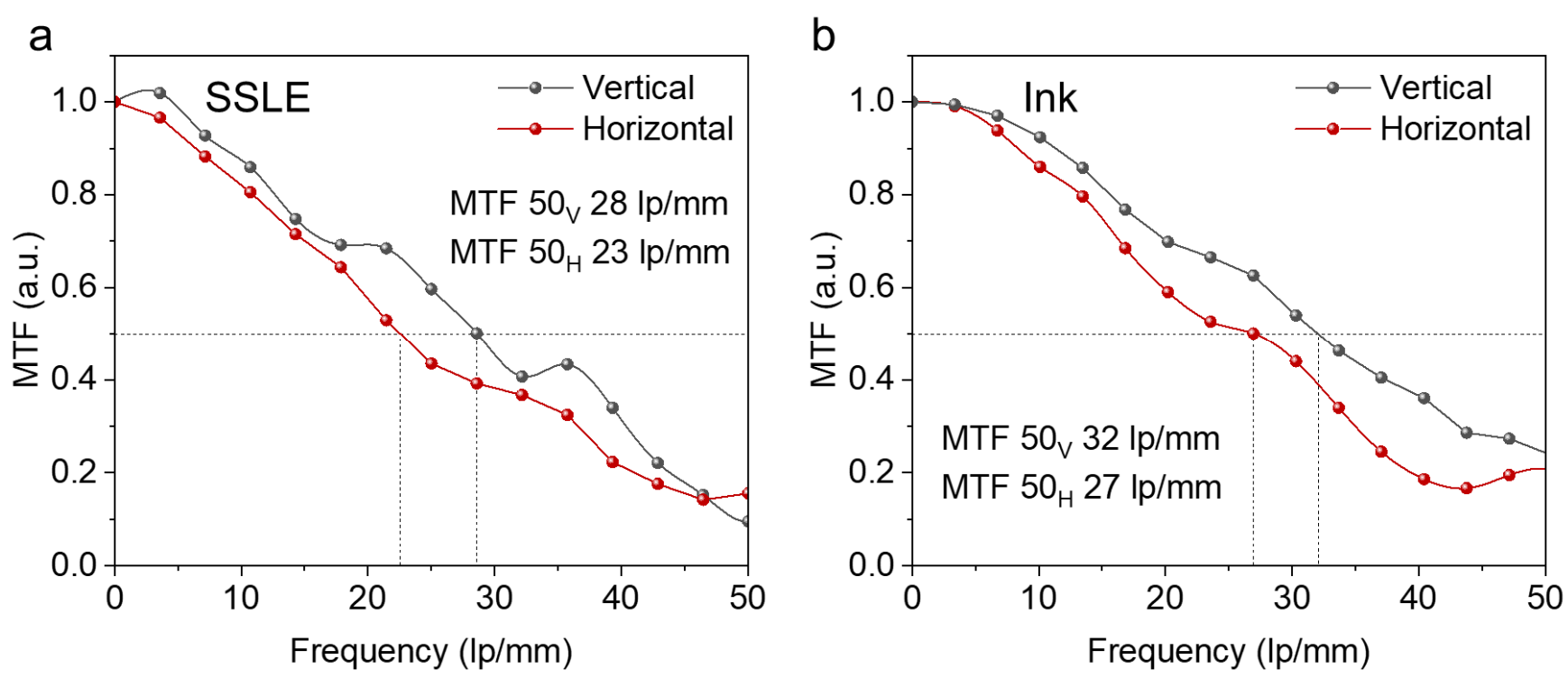

Figure S20. (a, b) Horizontal and vertical MTFs of the QD imager, SSLE- (a) and ink-based devices (b).

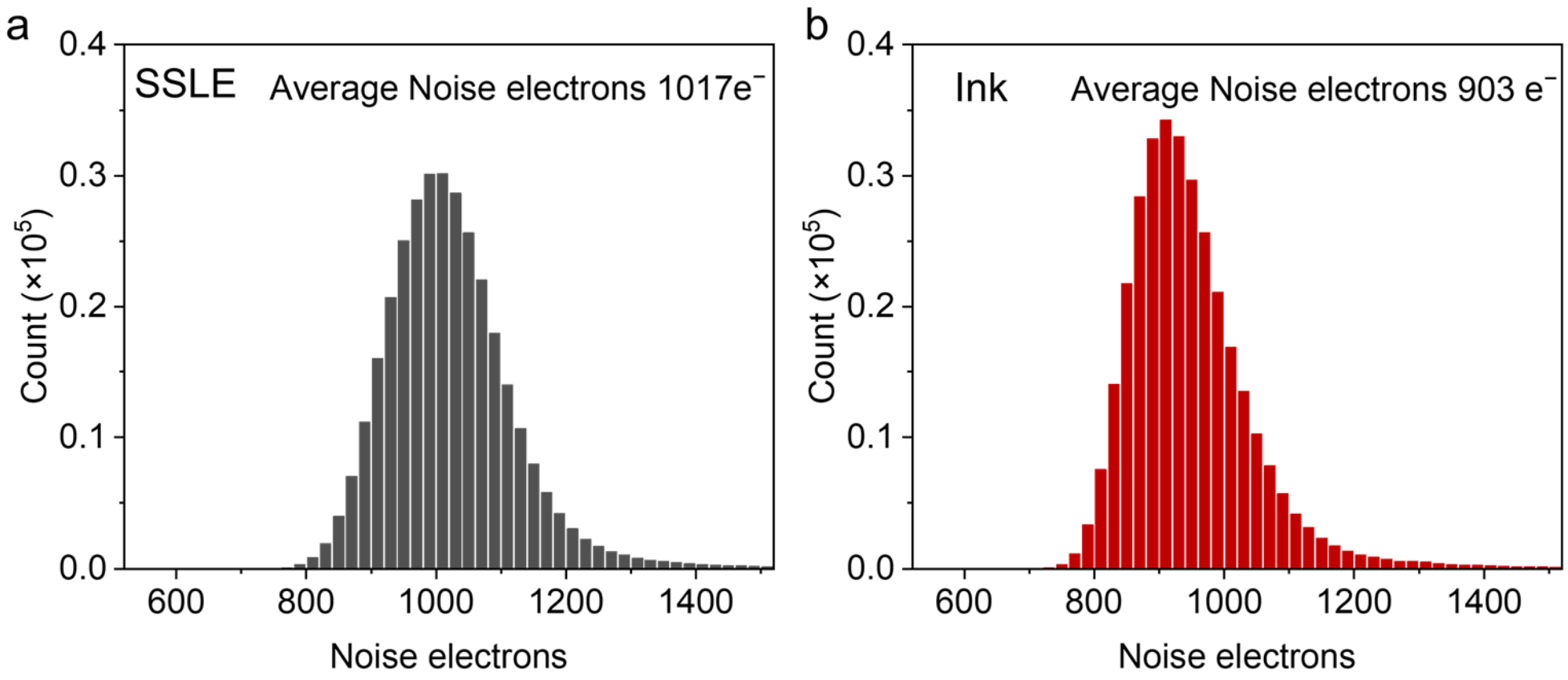

Figure S21. Statistical distributions of noise electrons for SSLE- (a) and ink-based devices (b).

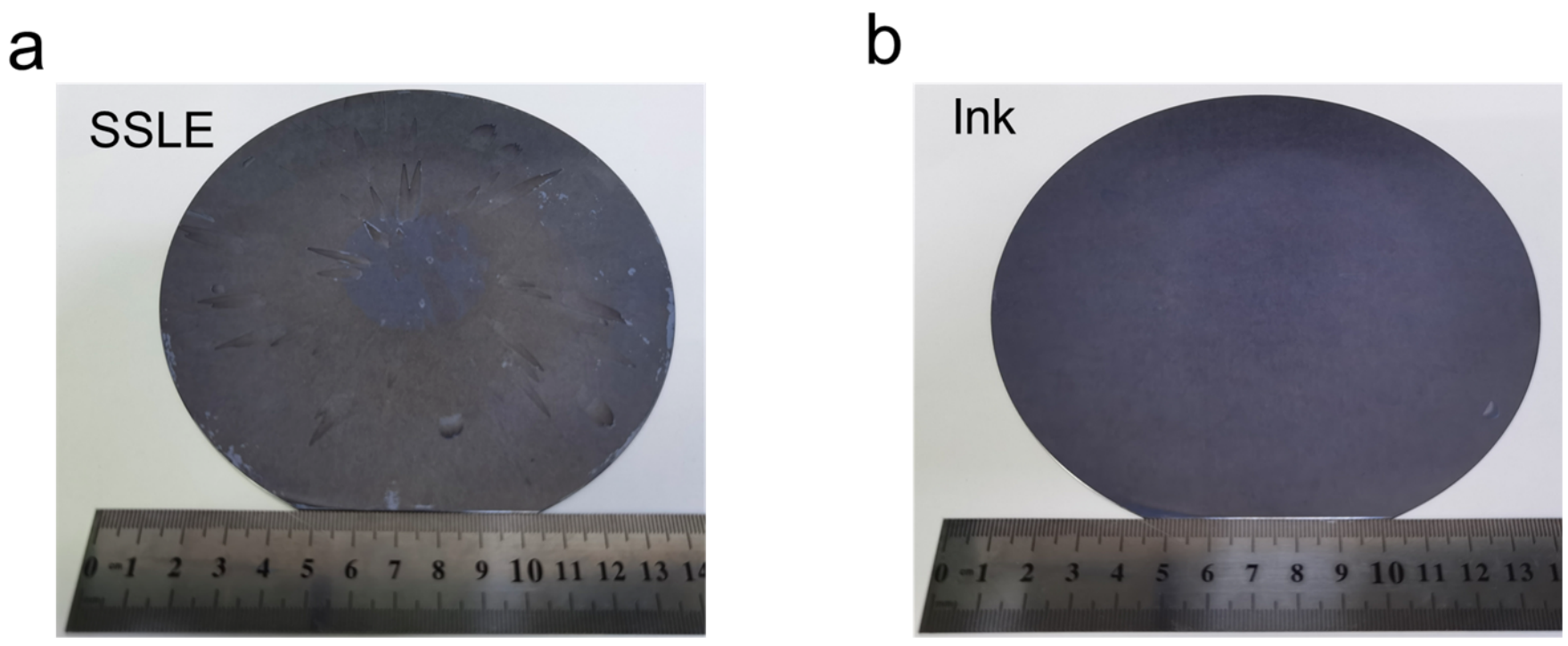

Figure S22. (a, b) Photo of SSLE- (a) and ink-based films (b) on a 6-inch Si wafer.

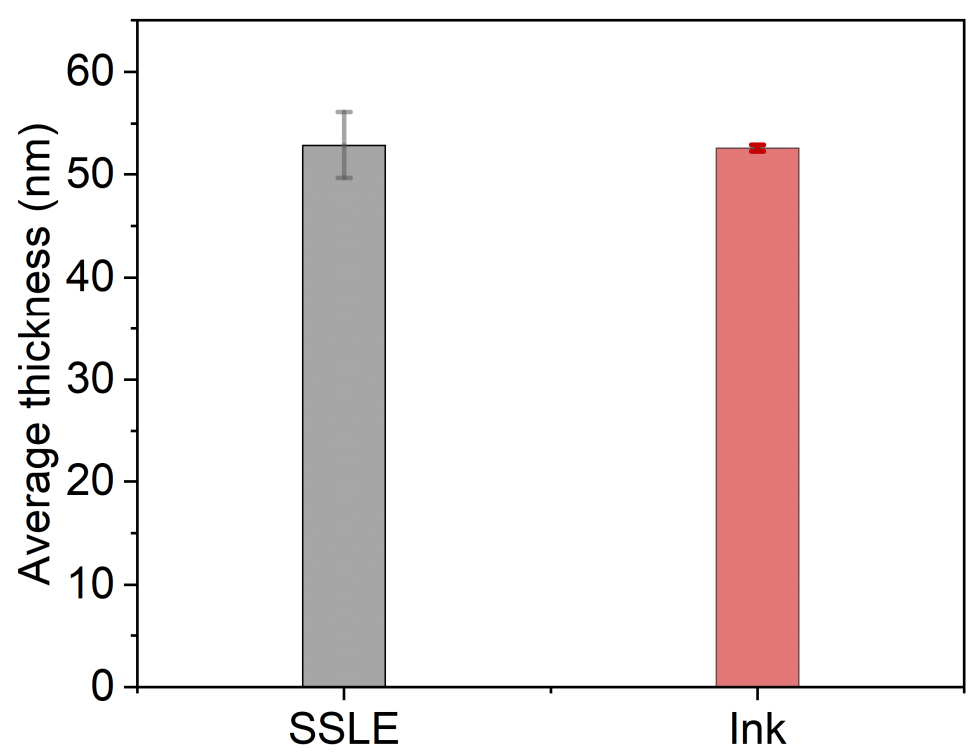

Figure S23. The average thickness of SSLE- and ink-based films on the 6-inch wafer.

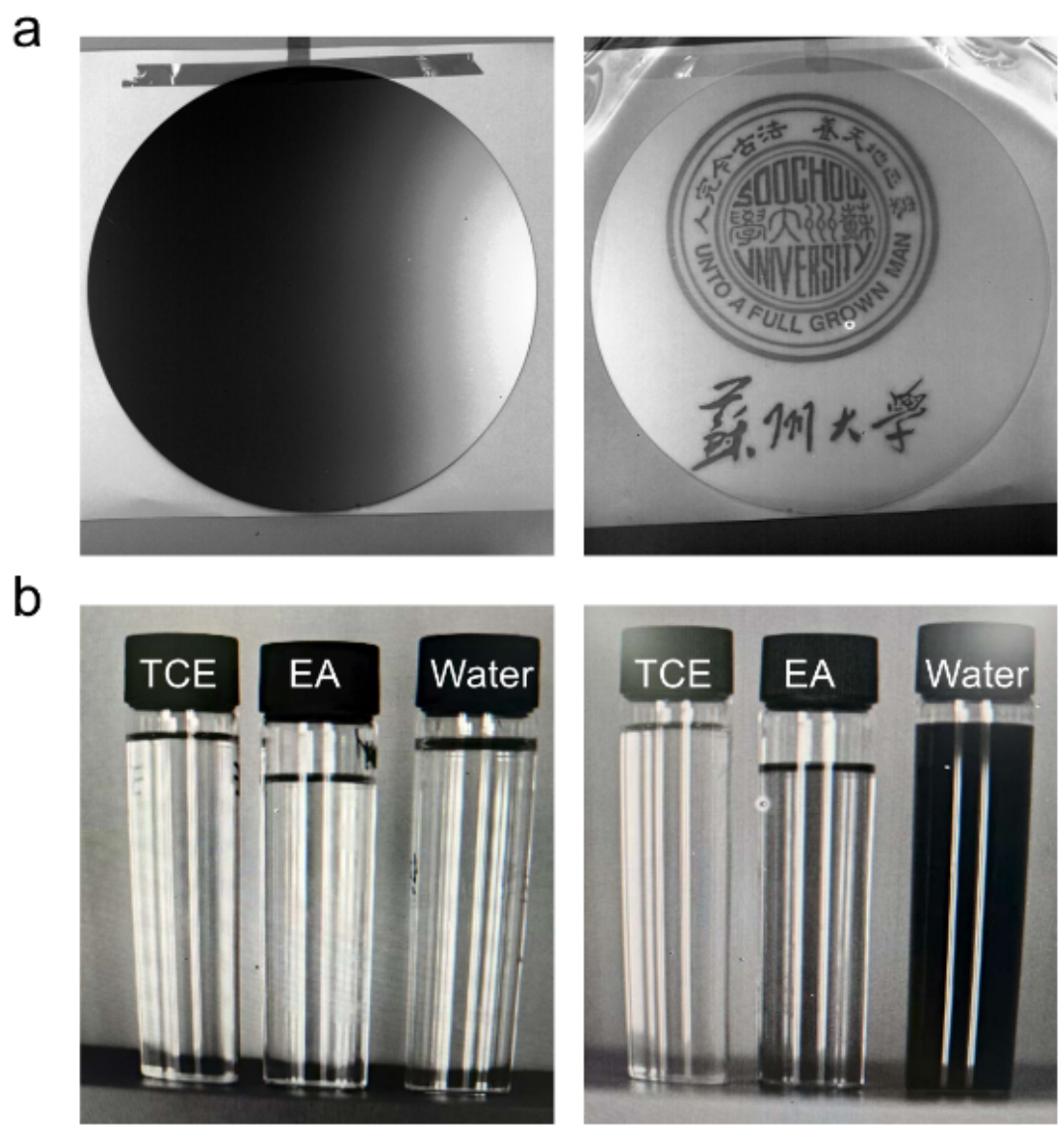

Figure S24. (a) Visible (left) and infrared (right) images from a scene containing a silicon wafer and a white paper with the words "Soochow University" printed on it. (b) visible image (left) and infrared (right) images from a scene containing a silicon wafer and three vials with different solvents. All images are captured at room temperature.

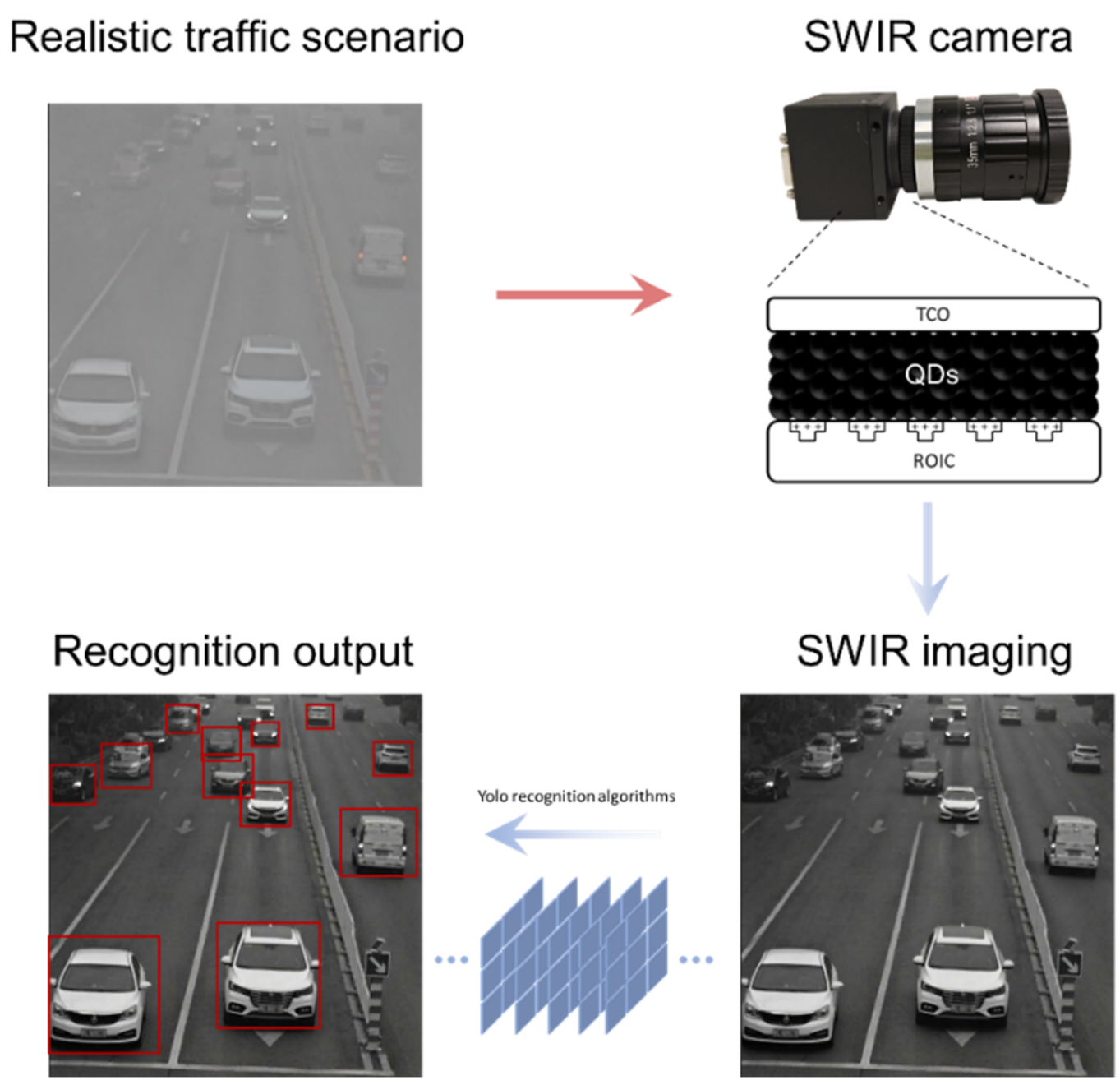

Figure S25. Schematic illustration of the QD-based SWIR imaging and vehicle-recognition pipeline.

REFERENCE

[1] Blöchl PE *Phys Rev B* 1994; **50:** 17953.

[2] Grimme S, Antony J, Ehrlich S *et al. J Chem Phys* 2010; **132:** 154104

[3] Hongzhiwei Technology, Device Studio, Version 2023A, China. Available online: https://cloud.hzwtech.com/web/product-service?id=6
